# Protective roles of apremilast via Sirtuin 1 in atherosclerosis

**DOI:** 10.1080/21655979.2022.2085390

**Published:** 2022-06-15

**Authors:** Dongkui Sui, Hua Yu

**Affiliations:** a Ultrasonography Department, the Affiliated Lianyungang Hospital of Xuzhou Medical University, the First People’s Hospital of Lianyungang, Lianyungang, Jiangsu, China; b Department of Cardiovascular Medicine, Affiliated Hospital of Jiujiang University, Jiujiang, Jiangxi, China

**Keywords:** Apremilast, atherosclerosis, SIRT1, inflammation, lipid accumulation

## Abstract

Atherosclerosis is an inflammatory disease with a high level of cholesterol in the blood. Apremilast is a new anti-inflammatory drug that possesses a potential anti-atherosclerosis effect. RT-qPCR and western blot were undertaken to assay the levels of Sirtuin 1 (SIRT1), oxidized low density lipoprotein receptor 1 (LOX-1), and CD36 molecule (CD36). Reactive oxygen species (ROS) levels were evaluated by 2’, 7’-dichlorodihydrofluorescein diacetate (DCFH-DA) staining, and Oil Red O staining was performed to show lipid accumulation. The result showed that apremilast treatment reduced the expression levels of pro-inflammatory factors and p-p65, as well as lipid accumulation. Meanwhile, triglyceride (TG), total cholesterol (TC) and free cholesterol (FC) levels declined in oxidized low density lipoprotein (ox-LDL)-treated macrophages. Mechanistically, apremilast targets SIRT1 and increases SIRT1 expression. The efficacy of apremilast on inflammatory response and lipid formation required the involvement of SIRT1. Additionally, apremilast treatment reduced scavenger receptors, LOX-1, and CD36 levels. These findings suggest the protective effects of apremilast via SIRT1 in atherogenesis and highlight the need for translational research from bench to bedside.

## Graphical Abstract

**Figure:**
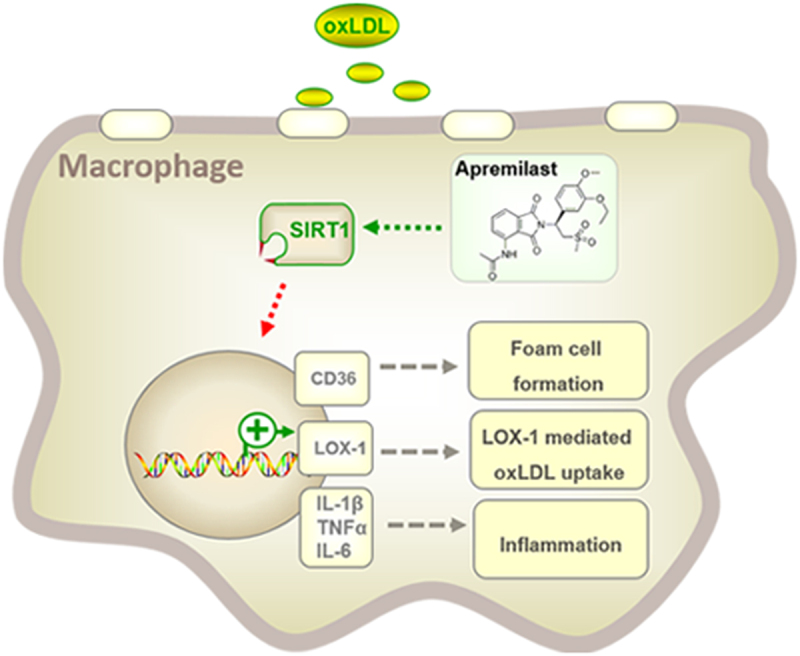

## Highlights

- Apremilast reduces the ox-LDL-induced inflammatory response
- Apremilast reduces ox-LDL-induced lipid accumulation
- SIRT1 signaling is involved in the regulation of apremilast

## Introduction

Atherosclerosis (AS) vulnerable plaque is an important pathological basis for the occurrence of acute cardiovascular disease. Macrophages are important promoters of the formation of AS-vulnerable plaques [1]. It is well known that AS plaque contains many foaming macrophages. During the development of the disease, cholesterol is phagocytosed by macrophages and deposited in the cells [2], forming foaming cells and promoting macrophages to secrete a large number of inflammatory cytokines which can aggravate local inflammation and lead to plaque damage and rupture, thereby promoting the progression of acute cardiovascular disease [3].

In a high-lipid environment, macrophages engulf oxidized low density lipoprotein (ox-LDL) and cholesterol crystals in their microenvironment. However, the body’s limited clearance ability leads to a large amount of lipid deposition in macrophages, which results in foaming, cell volume enlargement, and local lipid pools in blood vessels, and ultimately forming AS plaques [4–6]. Therefore, the present study used ox-LDL to treat macrophages to simulate the pathological process of AS. Recent studies have shown that apremilast can inhibit ox-LDL-induced endothelial barrier function injury by regulating Kruppel-like factor 6 [7], indicating that it has an important anti-atherosclerosis effect. However, whether apremilast can participate in ox-LDL-induced inflammatory release and foam formation of macrophages remains unknown. In systemic sclerosis, apremilast inhibits the recruitment and activation of macrophages and T cells, as well as the secretion of inflammatory factors [8], indicating that it could inhibit the injury of macrophages.

Therefore, this study aims to explore the impacts of apremilast on ox-LDL-treated macrophages. Whether apremilast can inhibit ox-LDL-induced macrophage inflammatory formation and foam formation is the point to be addressed.

## Method

### Cell culture

THP-1 cells were kept in culture in Roswell Park Memorial Institute (RPMI)1640 medium (Gibco, containing 5% fetal bovine serum, 50 U/mL penicillin and 50 μg/mL streptomycin). THP-1 cells at the logarithmic growth phase were seeded into 6-well plates (1 × 10^6^ cells/well) and phorbol myristate acetate (PMA, Beyotime, Shanghai, China) was added to induce macrophages [9]. Following 48-h cultivation at 37°C with 5% CO_2_, cells were employed for further assays.

### CCK8 assay

The macrophages were inoculated into 96-well plates (1 × 10^5^ cells/well) for 24 h before 100 μg/ mL ox-LDL and apremilast (2.5, 5, 10 μM) [10] were added. After the supplementation of CCK-8 reagent (10 μL/well) for 4 h, a microplate analyzer (Promega, WI, USA) was to estimate the optical density (OD) value at 490 nm [11].

### Reactive oxygen species (ROS) staining

The ROS levels were evaluated using the ROS Assay Kit (Beyotime, Shanghai, China) in line with the manufacturer’s guidance. 2’, 7’-Dichlorodihydrofluorescein diacetate (DCFH-DA) itself has no fluorescence and can freely cross the cell membrane. After entering the cell, DCFH-DA can be hydrolyzed by intracellular esterase to generate 2’, 7’-dichlorodihydrofluorescein (DCFH), but DCFH cannot penetrate the cell membrane so the probe can be easily loaded into the cell. ROS can oxidize the non-fluorescent DCFH to generate 2’, 7’-dichlorofluorescein (DCF) showing fluorescence. ROS level can be determined by measuring the fluorescence of DCF [12].

### Enzyme linked immunosorbent assay (ELISA)

Commercial ELISA kits were adopted for the determination of tumor necrosis factor (TNF)-α (cat. no. PT518), interleukin (IL)-1β (cat. no. PI305), and IL-6 (cat. no. PI330) levels strictly based on manual provided by the supplier (Beyotime, Shanghai, China) [13].

### Real time quantitative PCR (RT-qPCR) assay

Macrophages in each group (the control, ox-LDL, ox-LDL + Apremilast + (EX527)) were collected and the total RNA of macrophages was extracted using a total RNA extraction reagent (Tiagen Biochemical Technology, Beijing) following the standard procedures of kits. The RNA was synthesized into cDNA adopting M-MLV First Strand Kit (Invitrogen). In a 7500 quantitative PCR instrument from Applied Biosystems, the target gene was amplified with the application of the SYBR Green Master Mix (Beijing Baiao Laibo Technology Co., Ltd.). The calculation of gene expression was done using the 2^−ΔΔCt^ method, with GAPDH serving as endogenous control [14]. Primers are included in Table 1.

**Table 1.** Primer sequences

Gene	Sequence (5’→ 3’)
TNF-α	Forward: AGAACTCACTGGGGCCTACA
	Reverse: GCTCCGTGTCTCAAGGAAGT
IL-1β	Forward: GGCTGCTCTGGGATTCTCTT
	Reverse: ATTTCACTGGCGAGCTCAGG
IL-6	Forward: GGTCCAGTTGCCTTCTCCCTG
	Reverse: GCCCATGCTACATTTGCCG
SIRT1	Forward: CTACTGGCCTGAGGTTGAGG
	Reverse: GGACGGAGGAAAAGAGCGAA
LOX-1	Forward: GCTGGGCATGCAATTATCCC
	Reverse: TGGATGAAGTCCTGAACAATTTGC
CD36	Forward: GTTGAGAGCCTGTGCCTCAT
	Reverse: AGAAGAGCTTGCTTTCGGAGA
GAPDH	Forward: GACTCATGACCACAGTCCATGC
	Reverse: AGAGGCAGGGATGATGTTCTG

### Western blotting assay

Macrophages from each group were collected and lysed using RIPA lysis buffer, followed by protein quantification utilizing the bicinchoninic acid method. 10 μg total protein was subjected to sodium dodecyl sulfate polyacrylamide gel electrophoresis (SDS-PAGE) for separation and moved to the polyvinylidene difluoride (PVDF) membrane. 5% defatted milk powder in 0.5% TBST was prepared to block the membranes for 2 h. Primary antibodies against p-p65 (ab194926, 1: 1000, Abcam), p65 (ab16502, 1:2000, Abcam), SIRT1 (ab189494, 1:1000, Abcam), LOX-1 (ab214427, 1: 1000, Abcam), CD36 (GTX100642, 1:500, GeneTex, Shanghai) and GAPDH (GTX 100118, 1:10000; GeneTex) were employed to incubate the membrane at 4°C overnight. The membrane was rinsed 3 times by TBST and cultivated with Goat anti-rabbit secondary antibody (ab205718, 1:5000, Abcam) at room temperature for 2 h. The visualization and analysis of protein blots were carried out employing an ECL (Amersham) and Image J (version 1.52; National Institute of Health) [15].

### Oil red O staining

The macrophages were seeded into 24-well plates with 1 × 10^5^ cells/well and treated with 100 μg/ mL ox-LDL for 48 h. After fixation employing 4% paraformaldehyde for 10 min, these cells were subjected to wash twice in distilled water and soaked in 60% isopropyl alcohol for 5 min. Next, the newly prepared Oil Red O was added [16]. After 30 min, lipids were visualized with the application of a microscope.

### The evaluation of triglyceride (TG), total cholesterol (TC) and free cholesterol (FC) levels

Macrophages were collected for the evaluation of levels of TG, TC, and FC using the corresponding kit based on the manufacturer’s directions (Beyotime, Shanghai, China) [17].

### Statistical analysis

All data analysis was generated based on SPSS 22.0 and was manifested in the form of mean ± standard deviation (SD). The experiment was independently held three times. One-way analysis of variance (ANOVA) was used for the comparison of mean values among multiple groups, followed by Turkey’s test [11]. A significance level of P < 0. 05 implied statistical difference.

## Result

Recent studies have shown that apremilast can inhibit ox-LDL-induced endothelial barrier function injury, indicating that it has an important anti-atherosclerosis effect. However, whether apremilast can participate in ox-LDL-induced inflammatory release and foam formation of macrophages remains unknown. Hence, the purpose of this study was to address the above-mentioned points. ROS levels were evaluated and Oil Red O staining was performed to show lipid accumulation. TG, TC, and FC levels were determined in ox-LDL-treated macrophages. Moreover, the involvement of SIRT1 in the efficacy of apremilast on inflammatory response and lipid formation was assessed.

## Apremilast attenuates ox-LDL-induced macrophage inflammatory response and lipid accumulation

**Figure 1.**
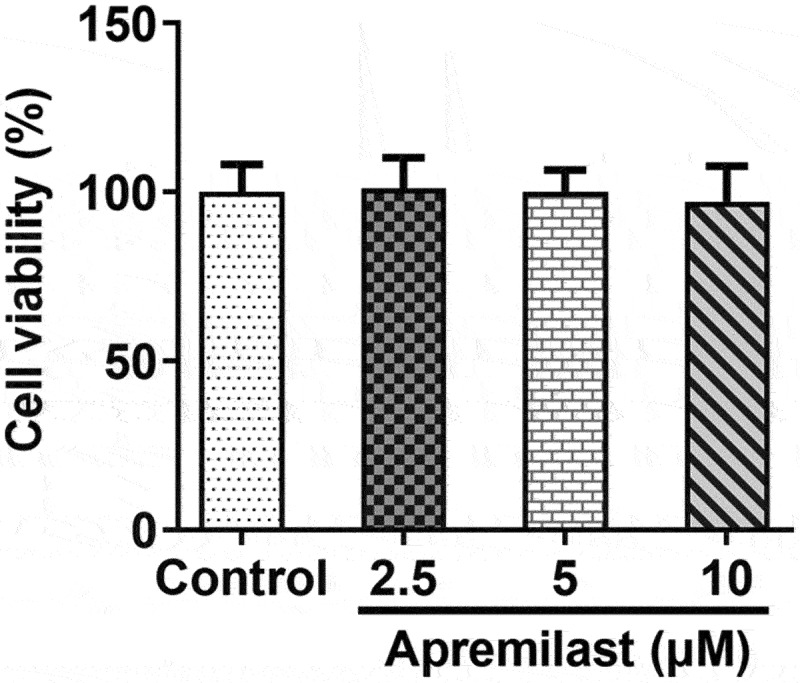
The effects of apremilast on cell activities on macrophages. CCK8 assay was performed to evaluate cell viability.

**Figure 2.**
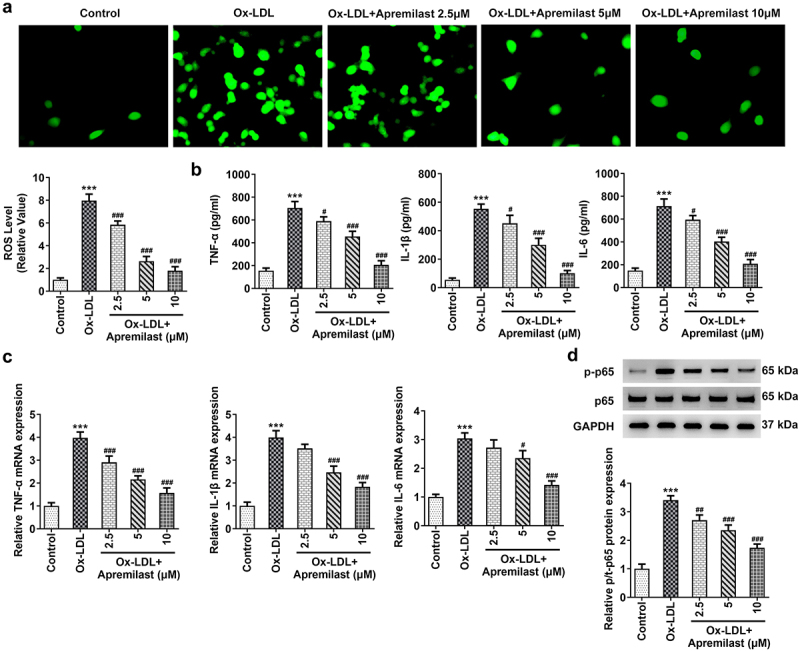
The effects of apremilast on inflammation in macrophages. (**a**) The representative images of ROS staining. (**b**) The expression levels of TNF-α, IL-1β, and IL-6 were measured using ELISA assay. (**c**) The expression levels of TNF-α, IL-1β, and IL-6 were measured using RT-qPCR. (**d**) The expression levels of p-p65 and p65 were measured using western blot. ***P < 0.001 vs. Control. ^#^P < 0.05, ^##^P < 0.01, ^###^P < 0.001 vs.

**Figure 3.**
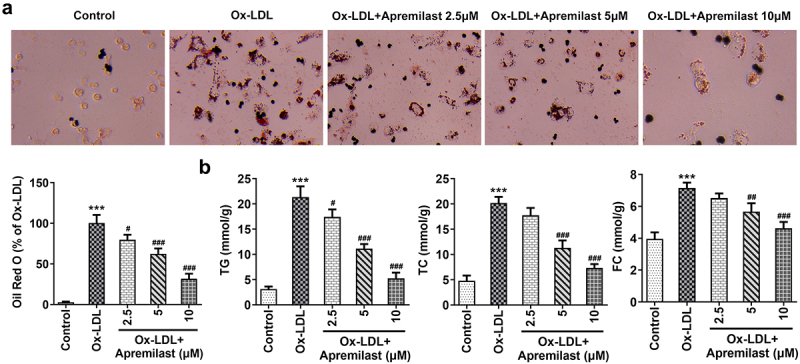
The effects of apremilast on lipid accumulation in macrophages. (**a**) The content of lipid droplets was estimated using Oil red O staining. (**b**) The measurement of TG, TC, and FC levels employed corresponding kits. ***P < 0.001 vs. Control. ^#^P < 0.05, ^##^P < 0.01, ^###^P < 0.001 vs. ox-LDL.

To investigate the impact of apremilast treatment on macrophages, macrophages were processed using apremilast at different concentrations. Cell viability remained unaffected (Figure 1(a)), suggesting that apremilast had no adverse effect. Next, we evaluated the efficacy of apremilast on inflammation and lipid accumulation in ox-LDL-treated macrophages. Apremilast treatment profoundly reduced the levels of ROS, TNF-α, IL-1β, IL-6, and p-p65 when compared with the ox-LDL group in a dose-dependent manner (Figure 2(a-d)). Furthermore, we found reduced lipid accumulation in ox-LDL-induced macrophage as assessed by oil red O staining (Figure 3(a)). Additionally, apremilast also decreases the TG, TC, and FC levels in ox-LDL-treated macrophages (Figure 3(b)). These results suggested that apremilast may reduce the inflammatory response and lipid accumulation during atherogenesis.

## Apremilast regulates SIRT1 to decrease ox-LDL-induced macrophage inflammatory response and lipid accumulation

**Figure 4.**
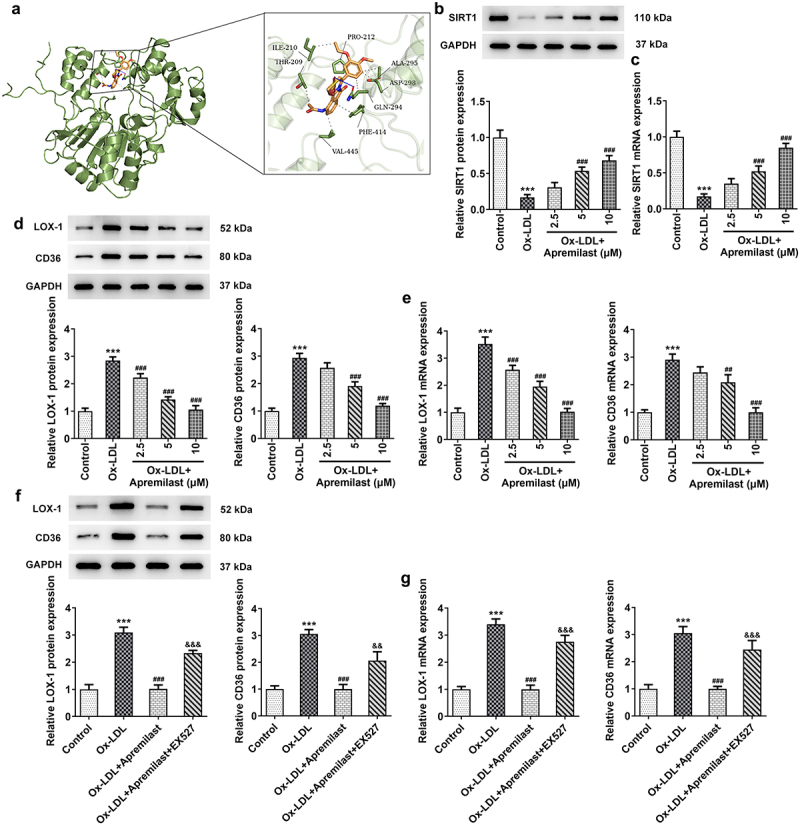
The effects of apremilast on SIRT1, LOX-1, and CD36 in macrophages. (**a**) The link between apremilast and SIRT1 was verified by the use of molecular docking. (**b**, **c**) The effects of Apremilast on the expression of SIRT1 were analyzed using RT-qPCR and western blot. (**d**, **e**) The effects of Apremilast on the expression of LOX-1 and CD36 were analyzed using RT-qPCR and western blot. ***P < 0.001 vs. Control. ^##^P < 0.01, ^###^P < 0.001 vs. ox-LDL. (**f**, **g**) The effects of EX527 addition on the expression of LOX-1 and CD36 were analyzed using RT-qPCR and western blot. ***P < 0.001 vs. Control. ^###^P < 0.001 vs. ox-LDL. ^&&^P < 0.01, ^&&&^P < 0.001 vs. ox-LDL + Apremilast.

**Figure 5.**
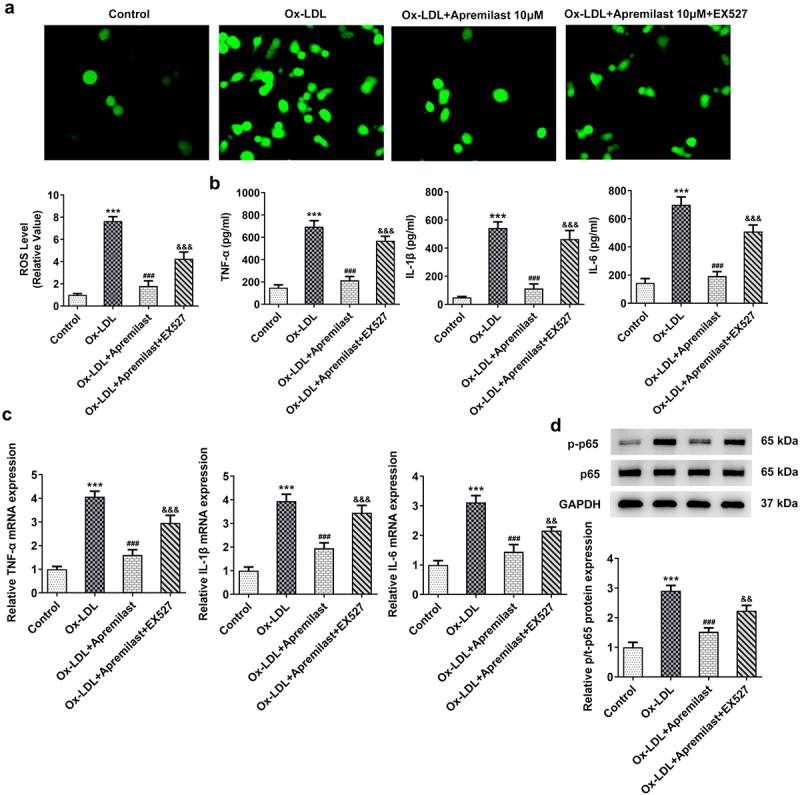
The effects of apremilast on inflammation were mediated by SIRT1 in macrophages. (**a**) The representative images of ROS staining. (**b**) The expression levels of TNF-α, IL-1β, and IL-6 were measured using ELISA assay. (**c**) The expression levels of TNF-α, IL-1β, and IL-6 were measured using RT-qPCR assay. (**d**) The expression levels of p-p65 and p65 were measured using western blot. ***P < 0.001 vs. Control. ^###^P < 0.001 vs. ox-LDL. ^&&^P < 0.01, ^&&&^P < 0.001 vs. ox-LDL + Apremilast.

**Figure 6.**
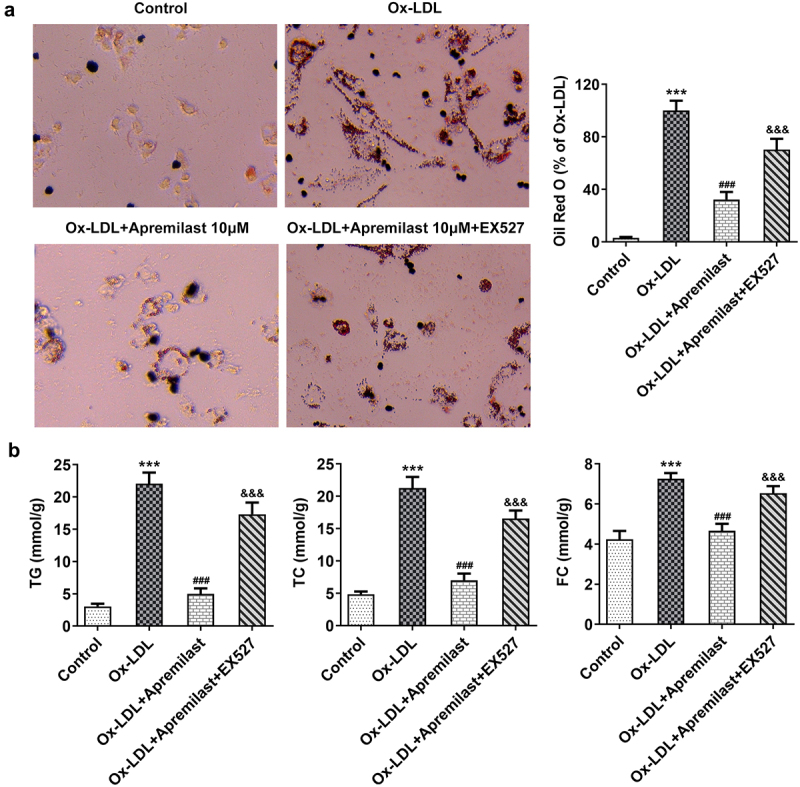
The effects of Apremilast on lipid accumulation were mediated by SIRT1 in macrophages. (**a**) The content of lipid droplets was estimated using Oil red O staining. (**b**) The measurement of TG, TC, and FC levels employed corresponding kits. ***P < 0.001 vs. Control. ^###^P < 0.001 vs. ox-LDL. ^&&&^P < 0.001 vs. ox-LDL + Apremilast.

The potentially beneficial role of apremilast in ox-LDL-induced macrophages prompted us to investigate its mechanism in ox-LDL-induced macrophages. To further investigate the potential molecular mechanism of apremilast, we performed molecular docking of apremilast. As shown in the result, apremilast could bind to SIRT1 through intermolecular forces (Figure 4(a)). Furthermore, apremilast treatment resulted in increased expression of SIRT1 which was decreased in ox-LDL-induced macrophages when compared with the control group (Figure 4(b-c)). We observed an increase in the production of LOX-1, a scavenger receptor for oxidized low-density lipoproteins (oxLDL) and CD36 after apremilast treatment in ox-LDL-induced macrophages (Figure 4(d-e)). EX527 addition, a SIRT1 inhibitor, resulted in the downregulation of LOX-1 and CD36 when compared with the co-treatment group of ox-LDL and apremilast (figure 4(f-g)). To better understand how apremilast affects inflammatory response and lipid accumulation, EX527 was added to evaluate the roles of SIRT1 in the efficacy of apremilast on the ox-LDL-induced macrophage. As shown in the result, EX527 resulted in the reverse effects of apremilast on ROS levels and inflammatory factors including TNF-α, IL-1β, and IL-6, as well as p-p65 in ox-LDL-induced macrophages (Figure 5(a-d)). We next tested whether the apremilast-mediated effects on lipid accumulation were mediated by SIRT1. EX527 addition significantly influences lipid accumulation and the levels of TG, TC, and FC which were decreased by apremilast treatment in ox-LDL-induced macrophages (Figure 6(a-b)).

## Discussion

The present study focused on the effects of apremilast on the inflammation and lipid accumulation induced by ox-LDL in macrophages. Apremilast treatment decreased inflammation as evidenced by decreased levels of the pro-inflammatory cytokines TNF-α, IL-1β, and IL-6, as well as decreased p-p65 levels, and lipid accumulation as assessed by oil red O staining and the detection of the levels of TG, TC, and FC. Apremilast is a selective inhibitor of phosphodiesterase 4 for the treatment of psoriasis and psoriatic arthritis, and the inhibition of which increases the levels of cyclic AMP, which leads to decreased pro-inflammatory cytokines [18]. The result is similar to a previous report finding that apremilast possess anti-atherogenic activities [7,19]. Further, the underlying mechanism by which apremilast could exert anti-atherogenic activities via affecting inflammation and lipid accumulation was verified to be related to SIRT1 in our work. The anti-inflammatory function of macrophages SIRT1 has been confirmed to be mediated by the NF-kB signaling pathway to downregulate the expression of various pro-inflammatory cytokines [20]. Apremilast might modulate NF-kB signaling pathway partly through suppressing p-p65 levels to suppressing the inflammatory response.

Apremilast was discovered to suppress the expressions of scavenger receptors LOX-1 and CD36, depending on the modulation of SIRT1. It was reported that SIRT1 inhibits the expression of Lox-1 to reduce uptake of ox-LDL, thereby preventing macrophage foam cell formation [21–24]. Therefore, LOX-1 and CD36 mediate the active uptake of ox-LDL into macrophages to lead to the formation of lipid droplets and foam cell formation. Additionally, a study demonstrated that SIRT1 is engaged in reverse cholesterol transport mechanisms [25], the dysfunction of which could result in abnormal accumulation of cholesterol in the cell which would induce foam cell formation and aggregate disease progression.

The present study observed that apremilast decreased lipid accumulation and the levels of TG, TC, and FC, this effect of which were profoundly blunted by the SIRT1 inhibitor, suggesting that apremilast may promote reverse cholesterol transport to decrease the excessive accumulation of cholesterol and therefore reduce macrophage foam cell formation via SIRT1. Nevertheless, there are several limitations to our study. Although our data suggest that apremilast promotes SIRT1 activation in ox-LDL-induced macrophages and that SIRT1 is required in macrophages in response to ox-LDL for apremilast-mediated the inhibition of inflammatory response and lipid accumulation, there could be the unidentified pathways involved in the process. Additionally, *in vivo* studies are still required for deeper exploration of the efficacy of apremilast against AS.

## Conclusion

Taken together, this study indicates that apremilast not only decreases the uptake of ox-LDL, but also promotes reverse cholesterol transport via SIRT1. Hence, repurposing apremilast for the treatment or prevention of AS would be feasible. The present study provides proof of the principle that therapies aimed at inhibiting inflammation and lipid accumulation could be an effective strategy for the treatment of AS. The role of apremilast in other aspects of AS and the deeper mechanism will be our future research directions.

## Supplementary Material

Supplemental Material
