# Supplementary figures and images for: Protective roles of apremilast via Sirtuin 1 in atherosclerosis
Source: Bioengineered. 2022 Jun 15;13(5):13872–81. doi: 10.1080/21655979.2022.2085390 (PMC9276050; doi:10.1080/21655979.2022.2085390)

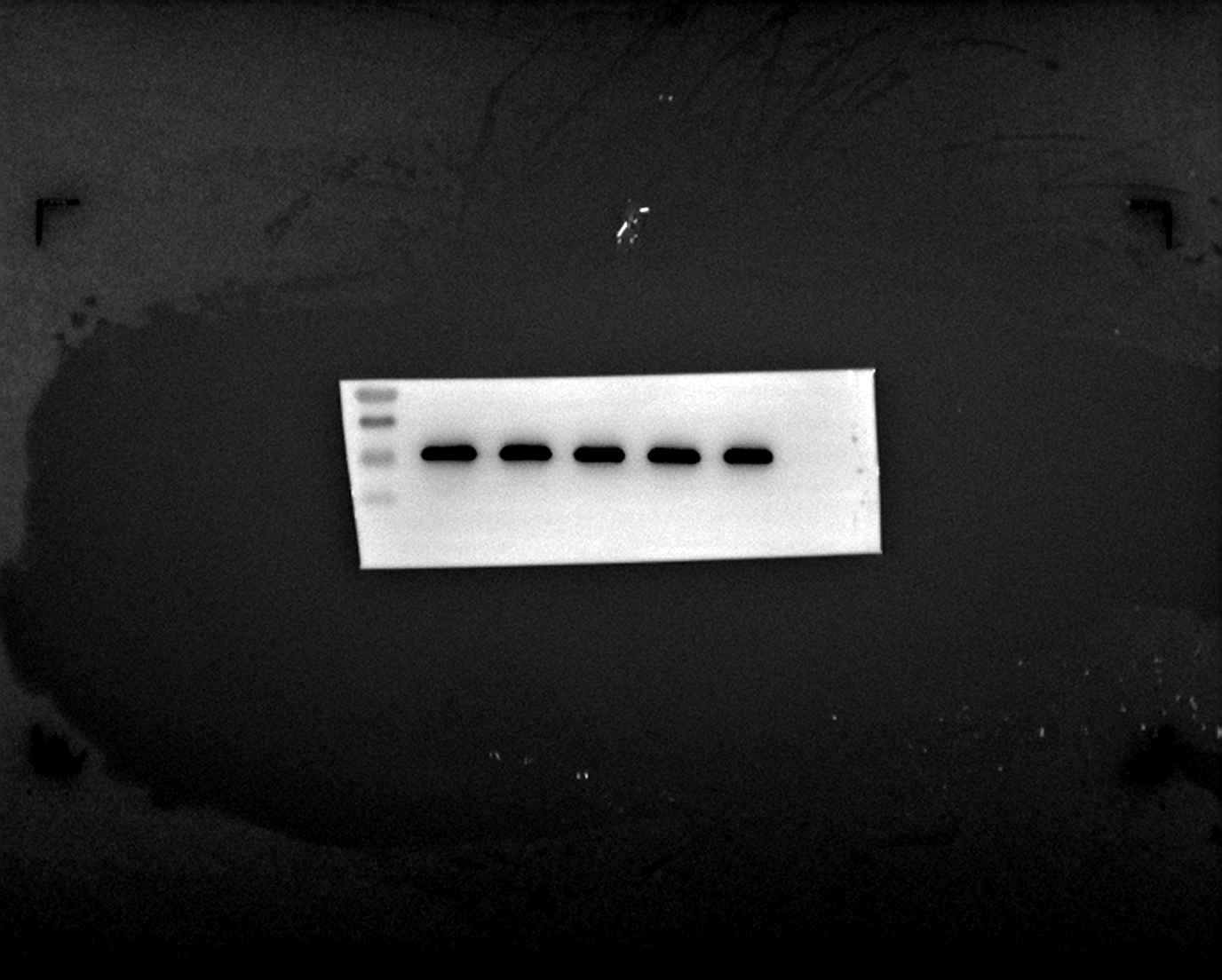

Supplement: Supplemental Material [file KBIE_A_2085390_SM9860.zip › Original Image/Blots Fig2D/GAPDH.Tif]

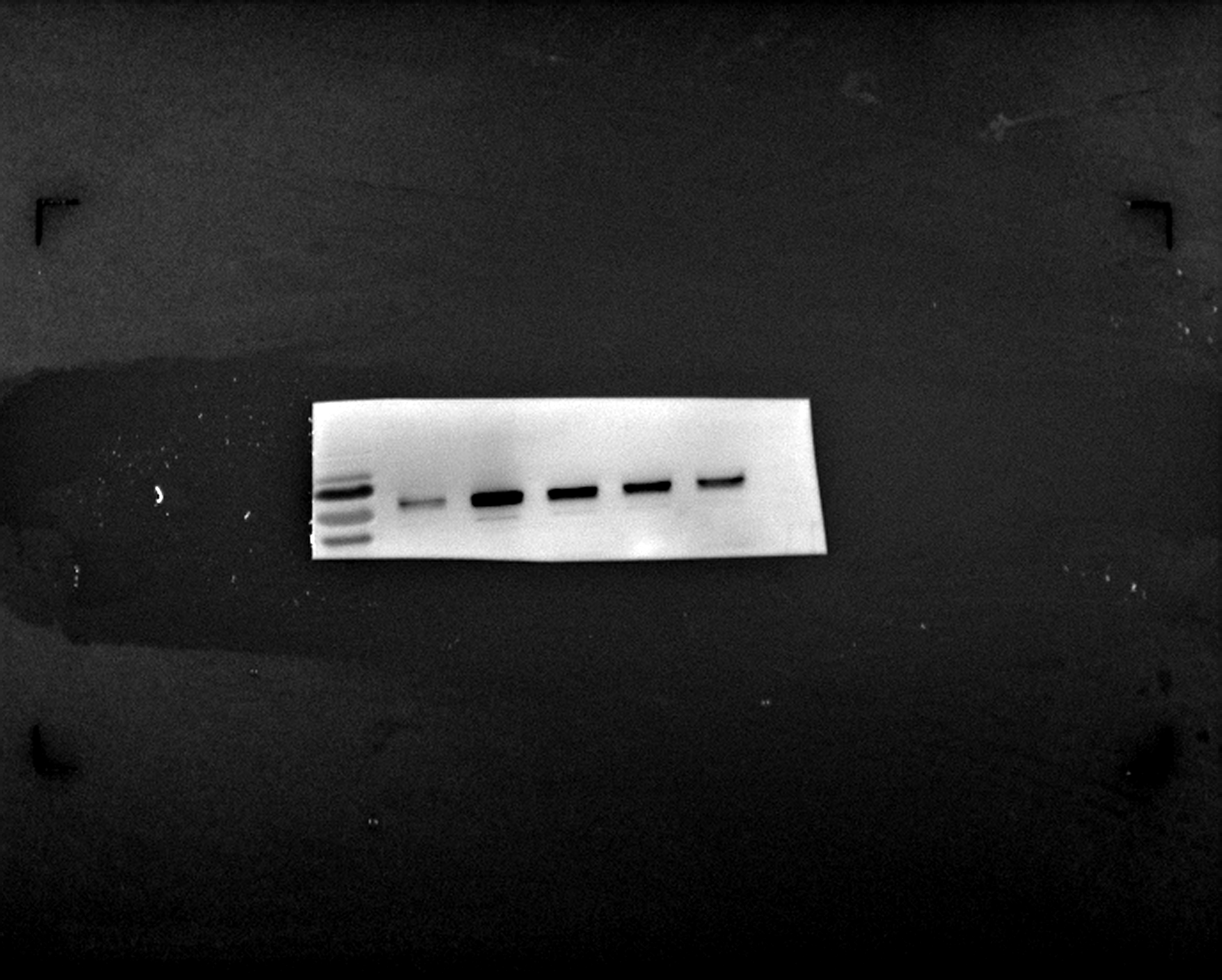

Supplement: Supplemental Material [file KBIE_A_2085390_SM9860.zip › Original Image/Blots Fig2D/p-p65.Tif]

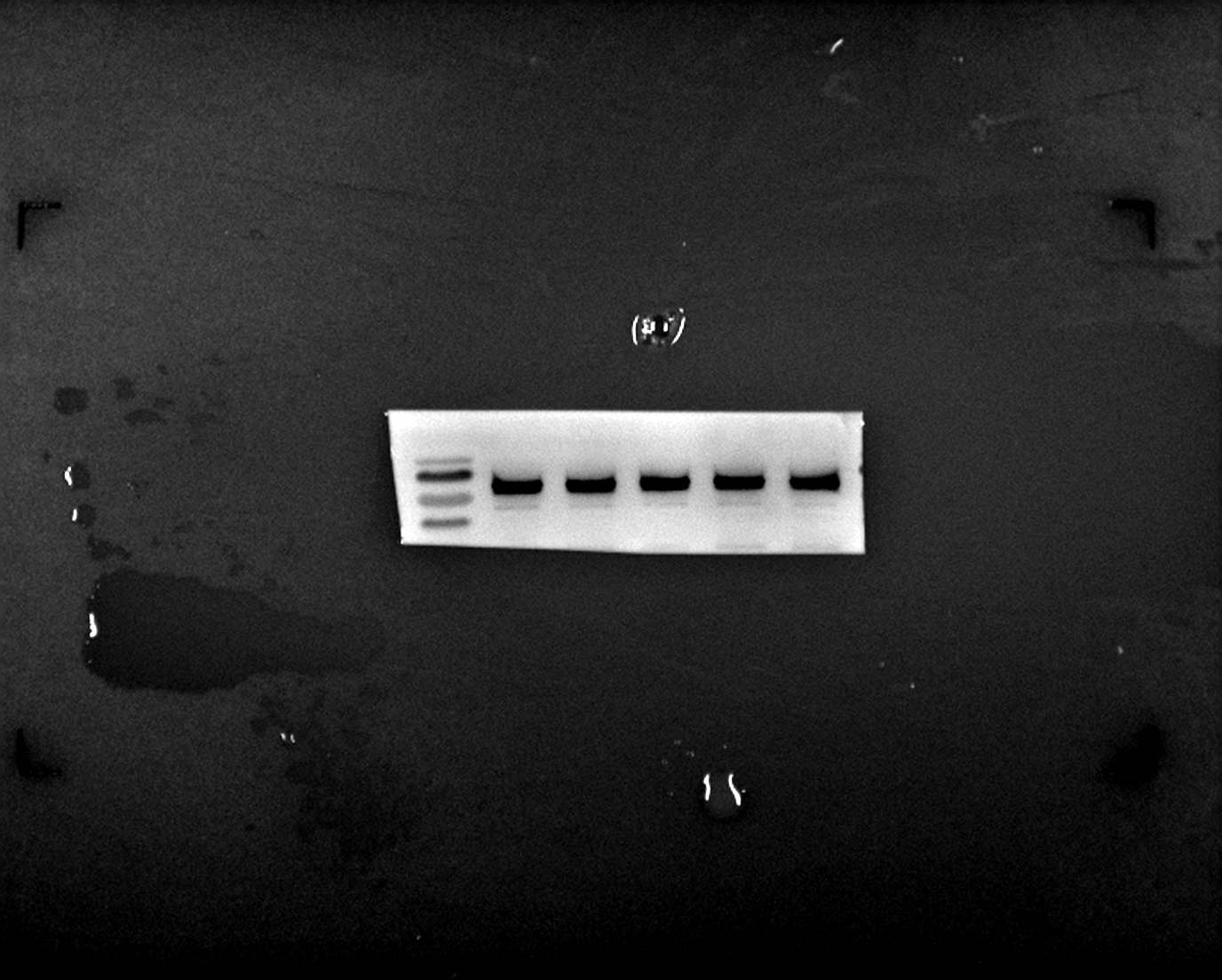

Supplement: Supplemental Material [file KBIE_A_2085390_SM9860.zip › Original Image/Blots Fig2D/p65.Tif]

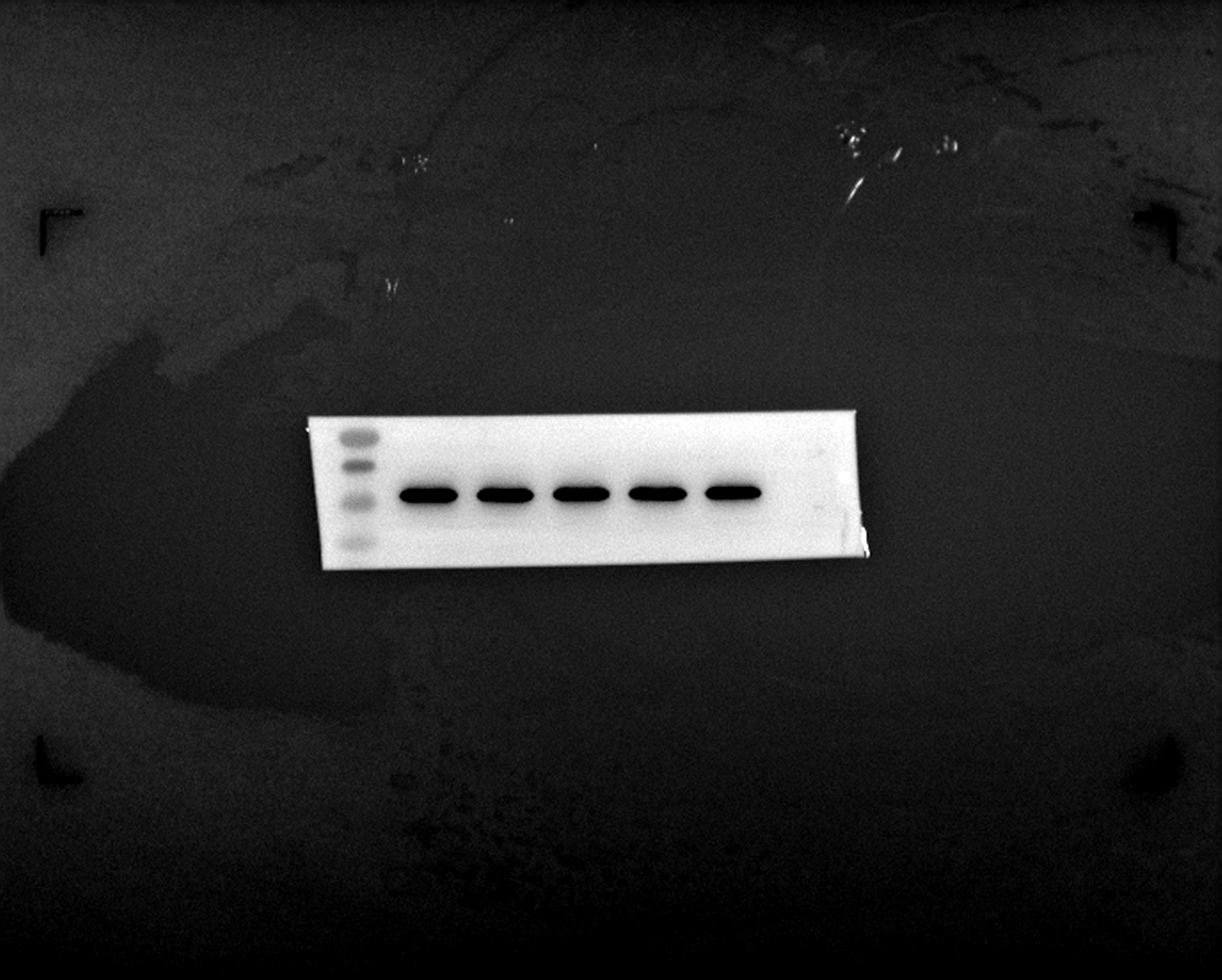

Supplement: Supplemental Material [file KBIE_A_2085390_SM9860.zip › Original Image/Blots Fig4B/GAPDH.Tif]

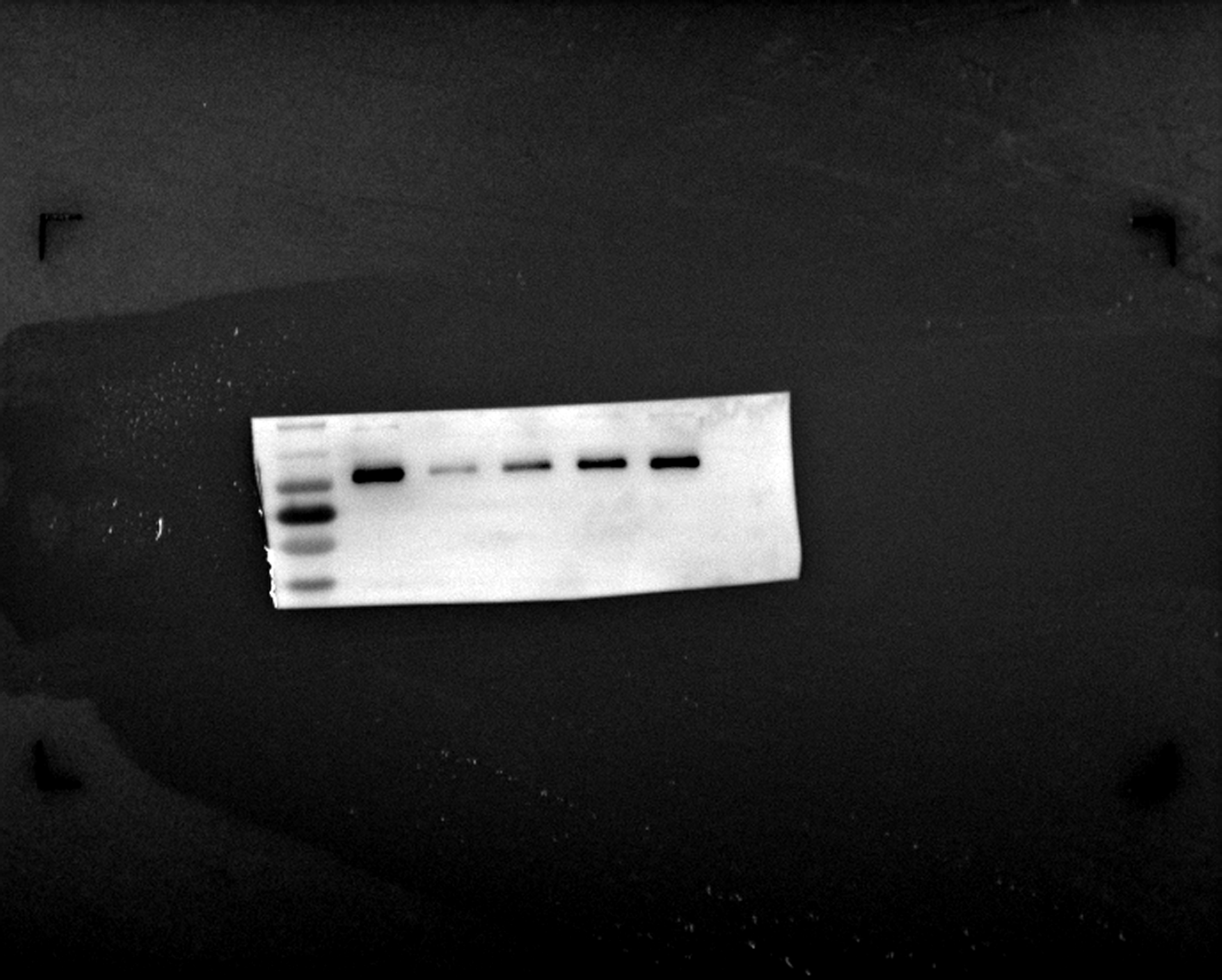

Supplement: Supplemental Material [file KBIE_A_2085390_SM9860.zip › Original Image/Blots Fig4B/SIRT1.Tif]

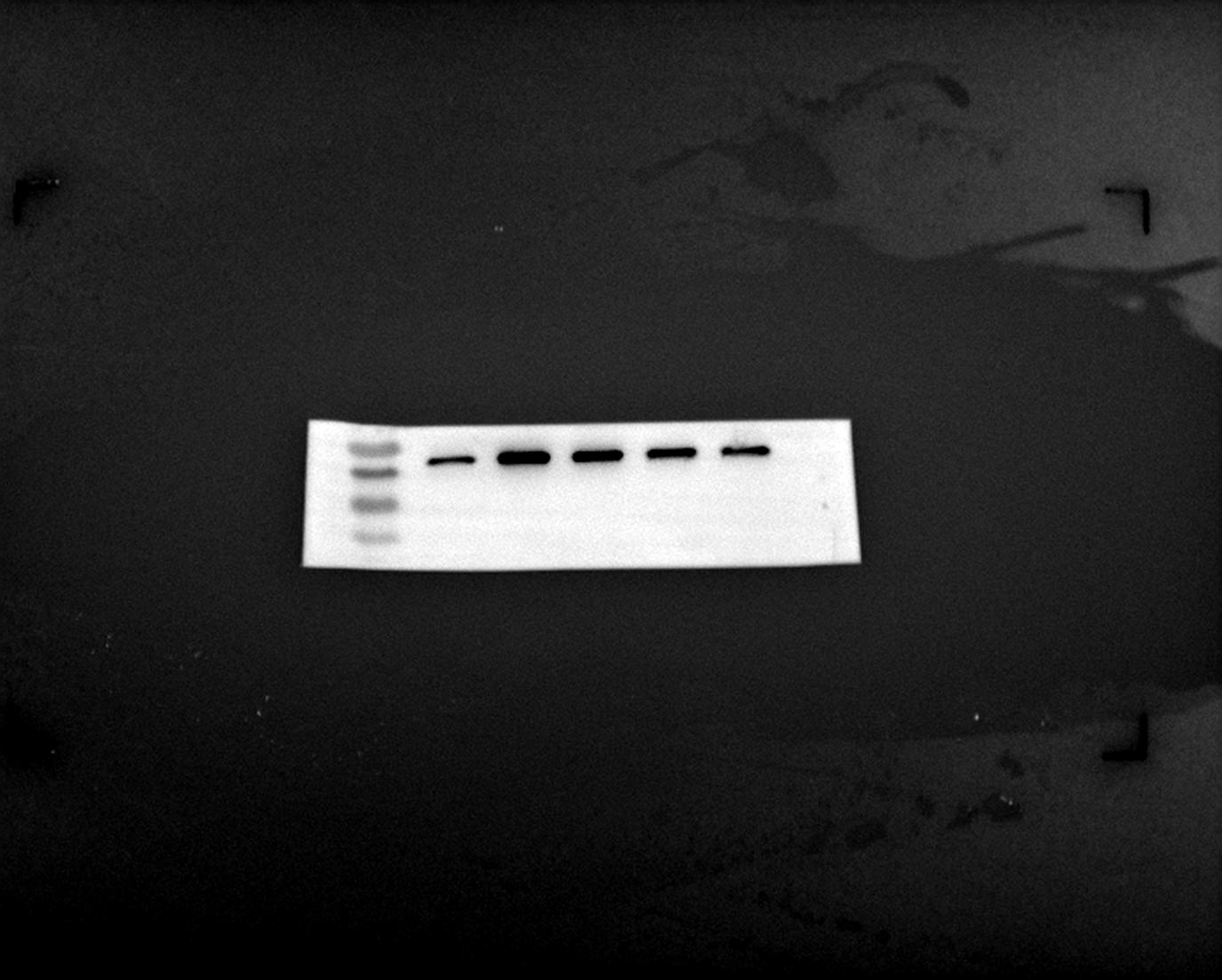

Supplement: Supplemental Material [file KBIE_A_2085390_SM9860.zip › Original Image/Blots Fig4D/CD36.Tif]

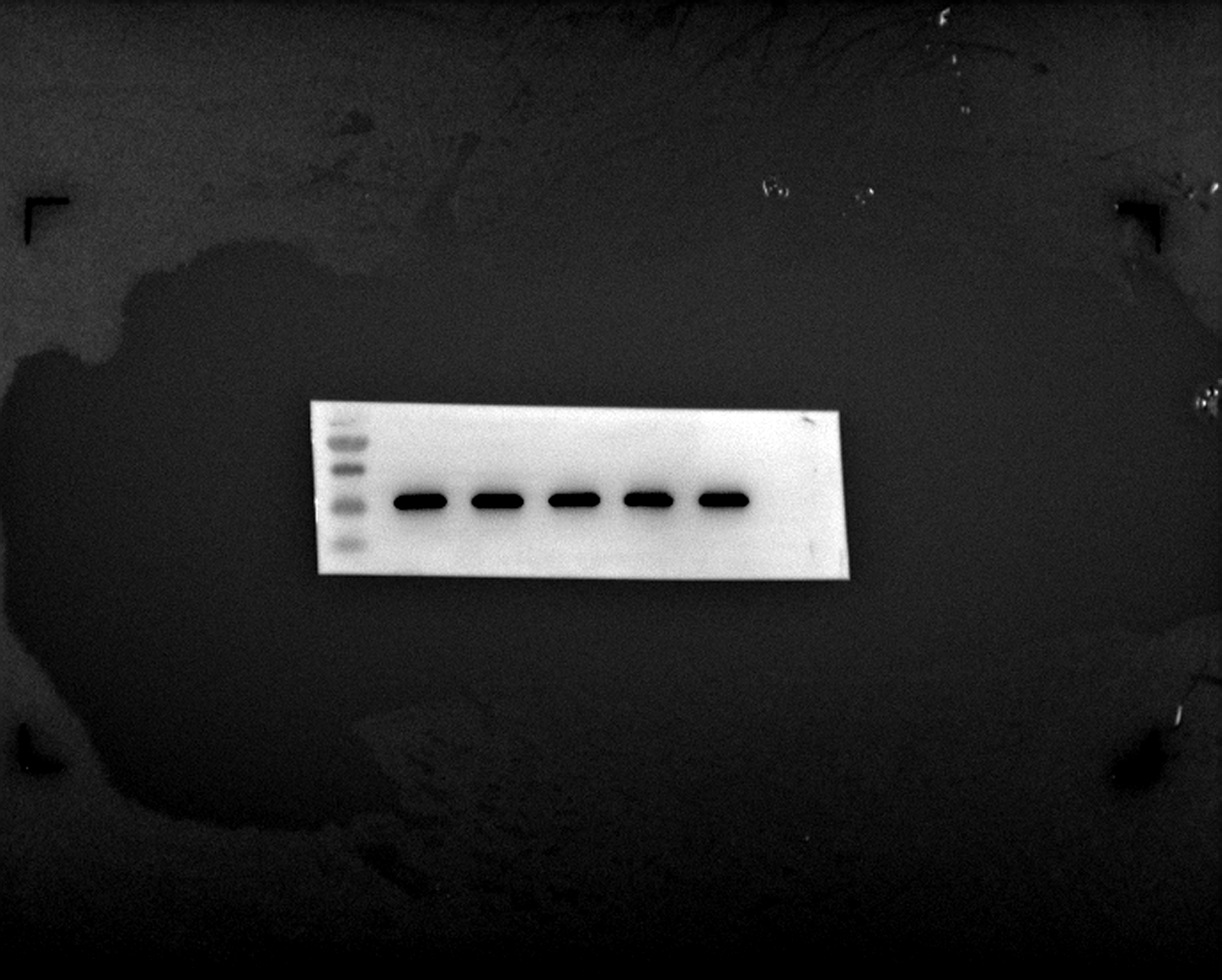

Supplement: Supplemental Material [file KBIE_A_2085390_SM9860.zip › Original Image/Blots Fig4D/GAPDH.Tif]

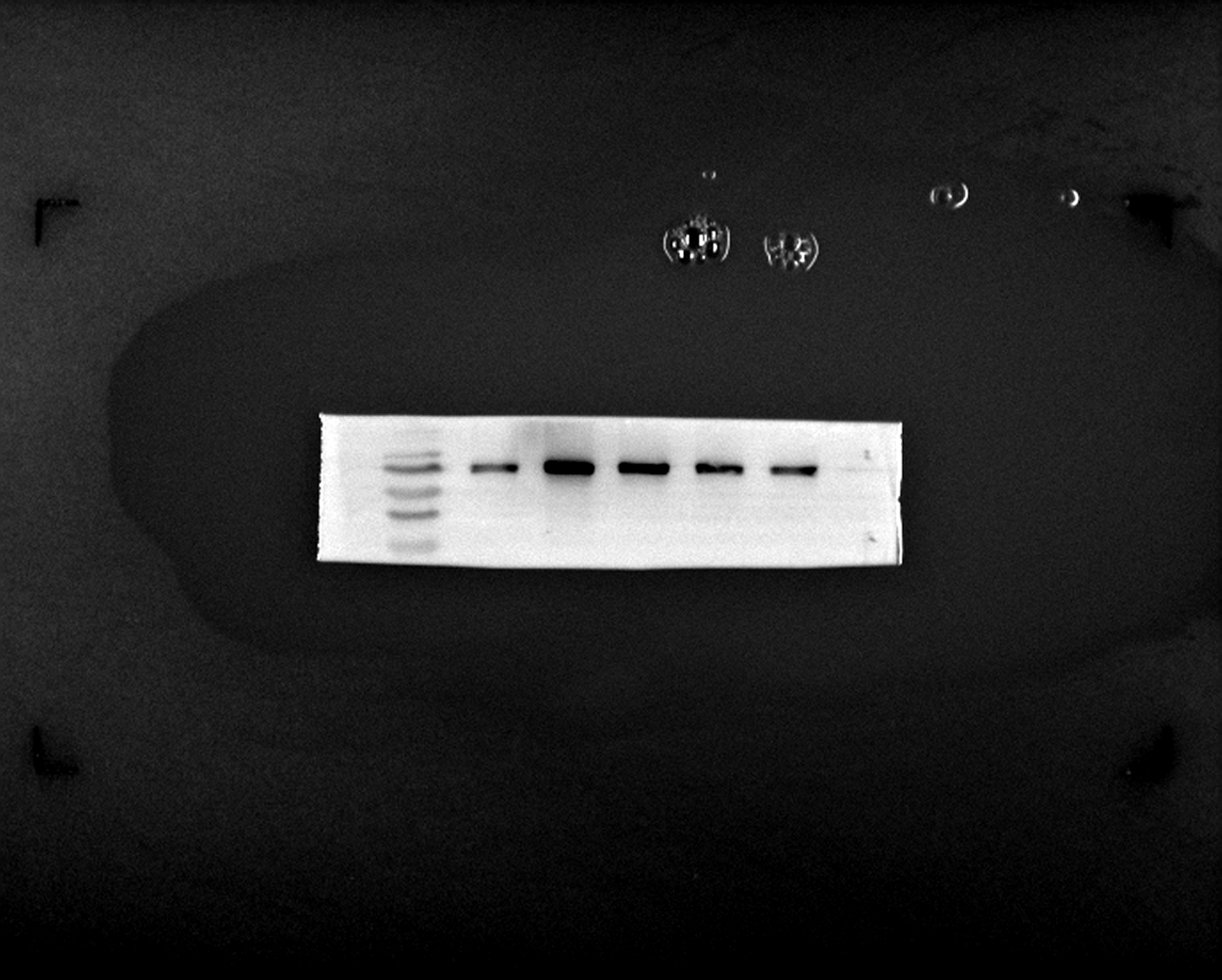

Supplement: Supplemental Material [file KBIE_A_2085390_SM9860.zip › Original Image/Blots Fig4D/LOX-1.Tif]

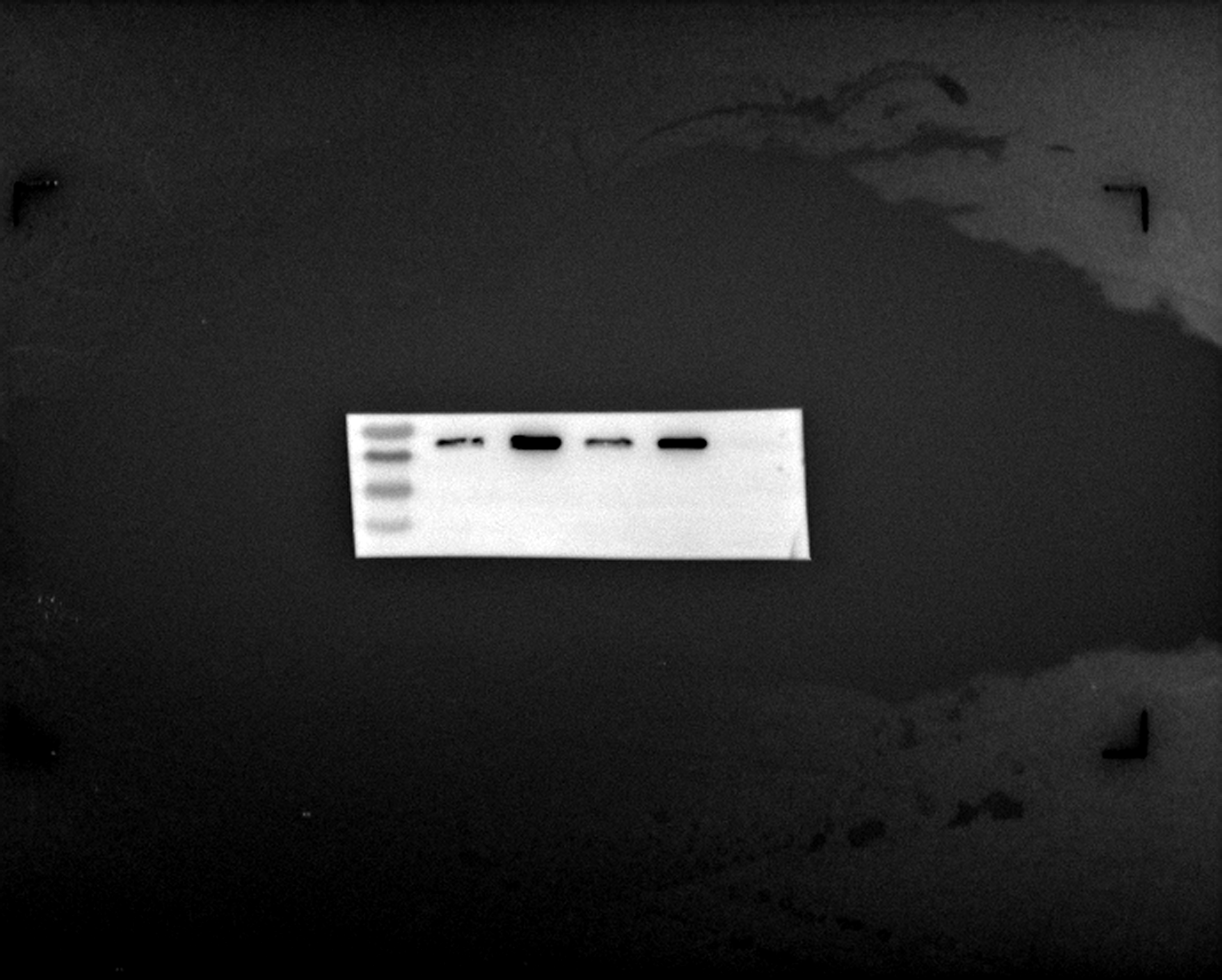

Supplement: Supplemental Material [file KBIE_A_2085390_SM9860.zip › Original Image/Blots Fig4F/CD36.Tif]

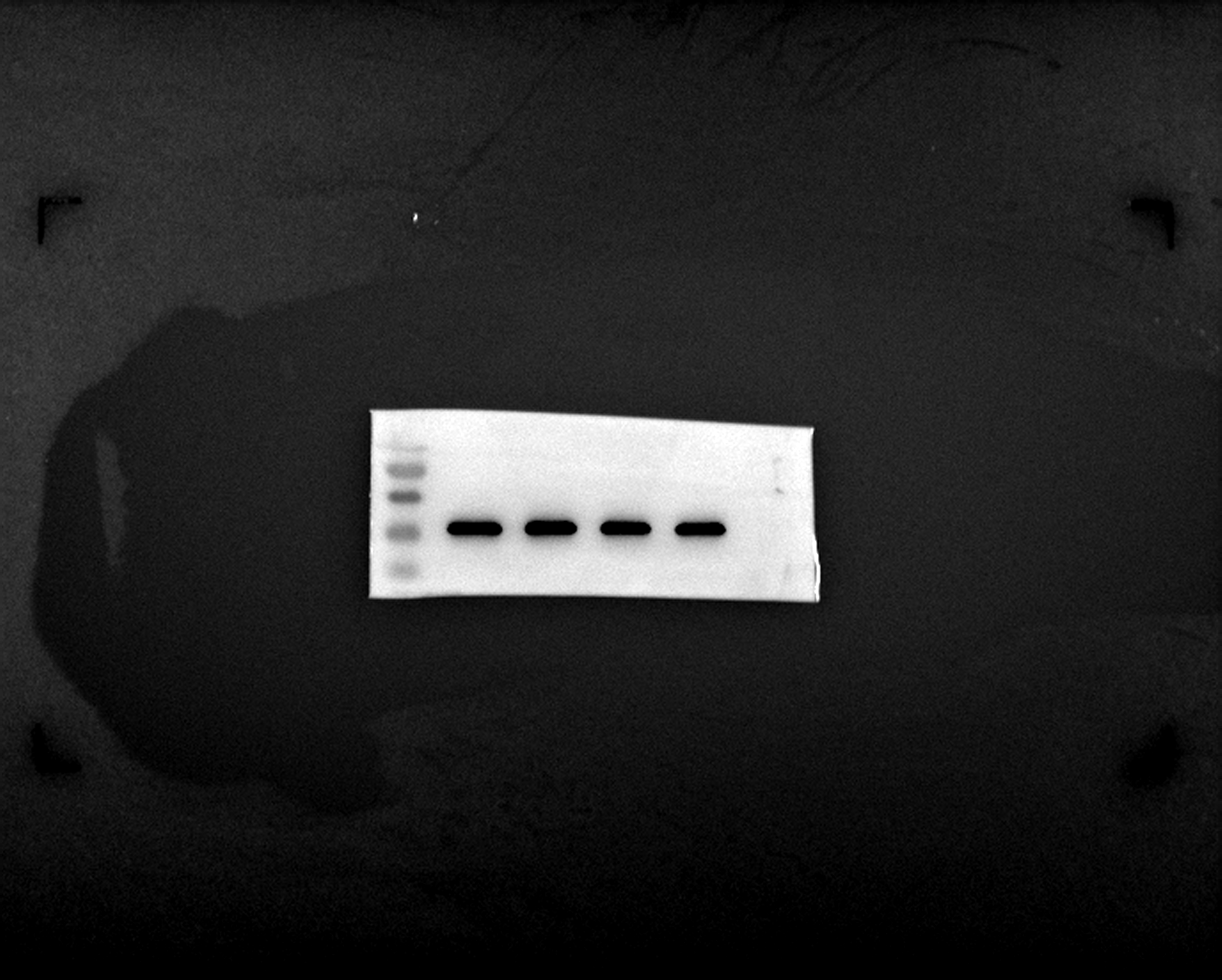

Supplement: Supplemental Material [file KBIE_A_2085390_SM9860.zip › Original Image/Blots Fig4F/GAPDH.Tif]

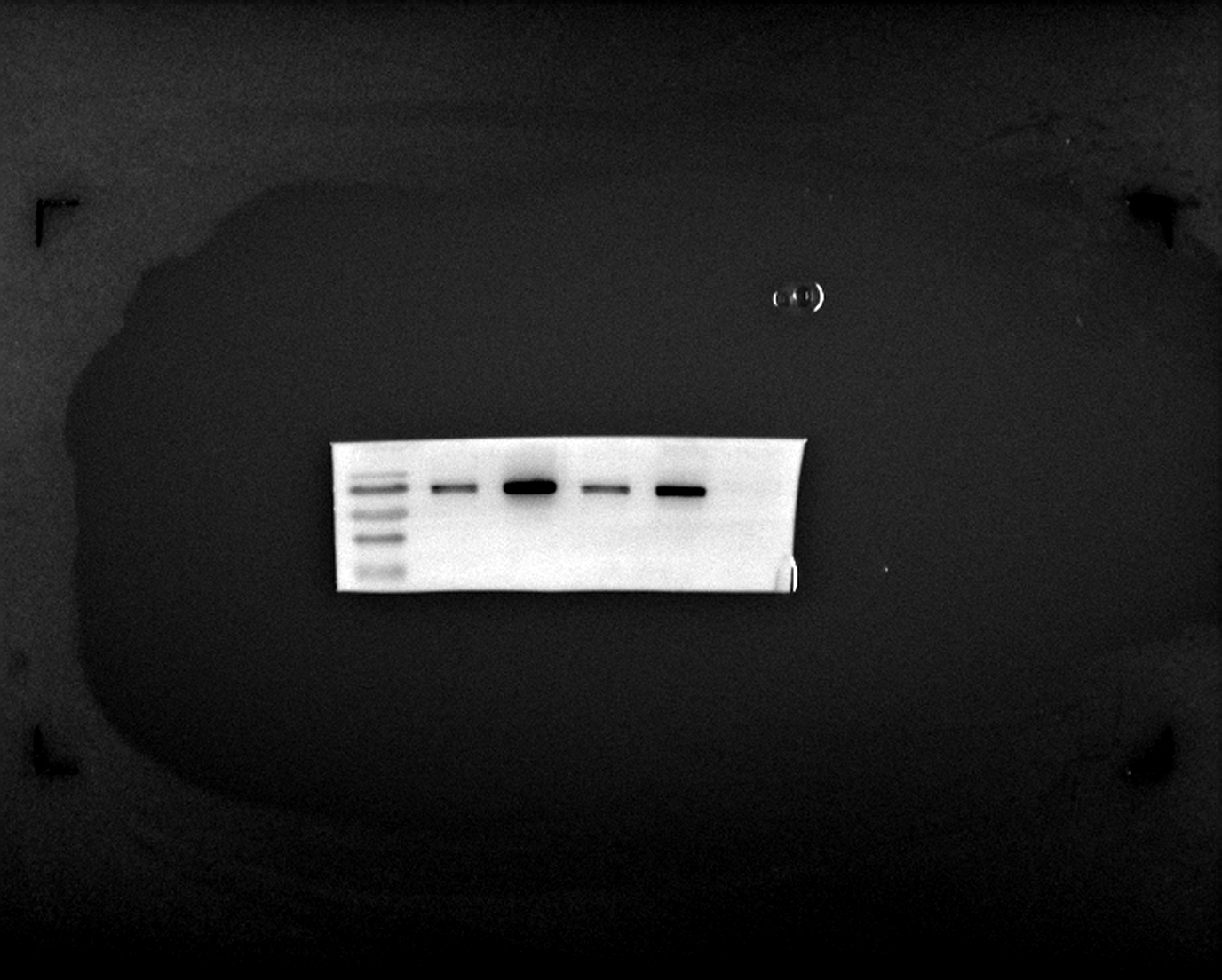

Supplement: Supplemental Material [file KBIE_A_2085390_SM9860.zip › Original Image/Blots Fig4F/LOX-1.Tif]

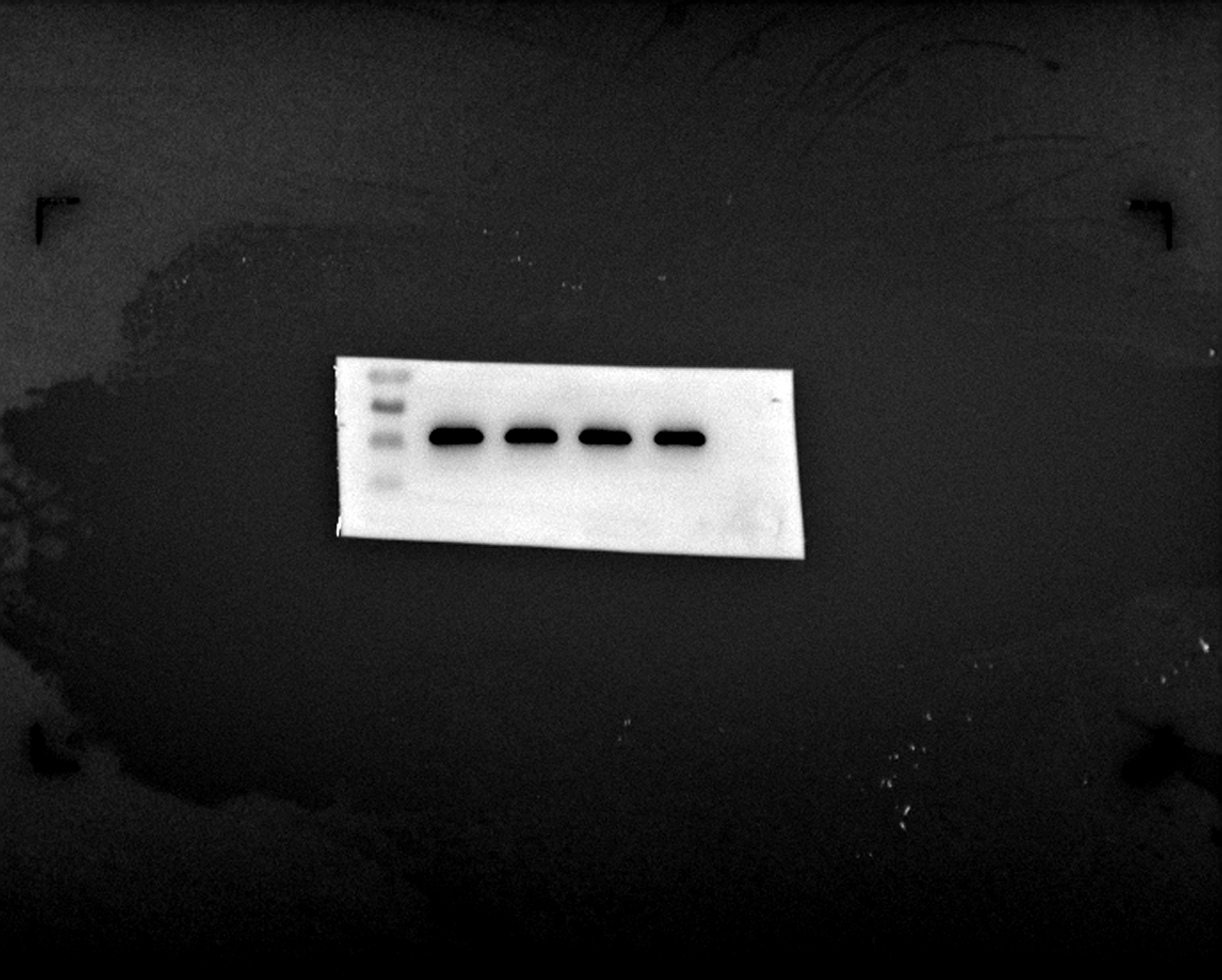

Supplement: Supplemental Material [file KBIE_A_2085390_SM9860.zip › Original Image/Blots Fig5D/GAPDH.Tif]

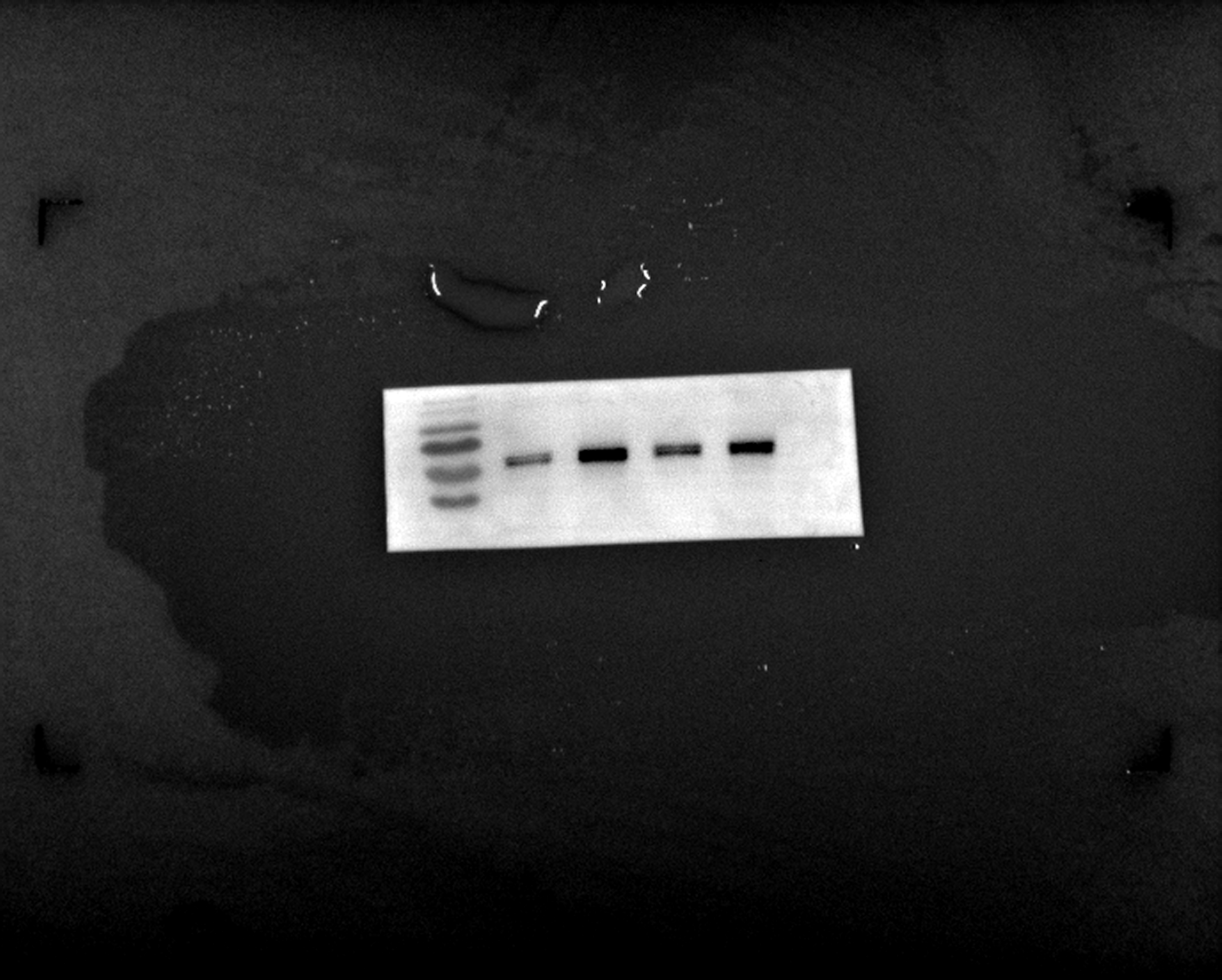

Supplement: Supplemental Material [file KBIE_A_2085390_SM9860.zip › Original Image/Blots Fig5D/p-p65.Tif]

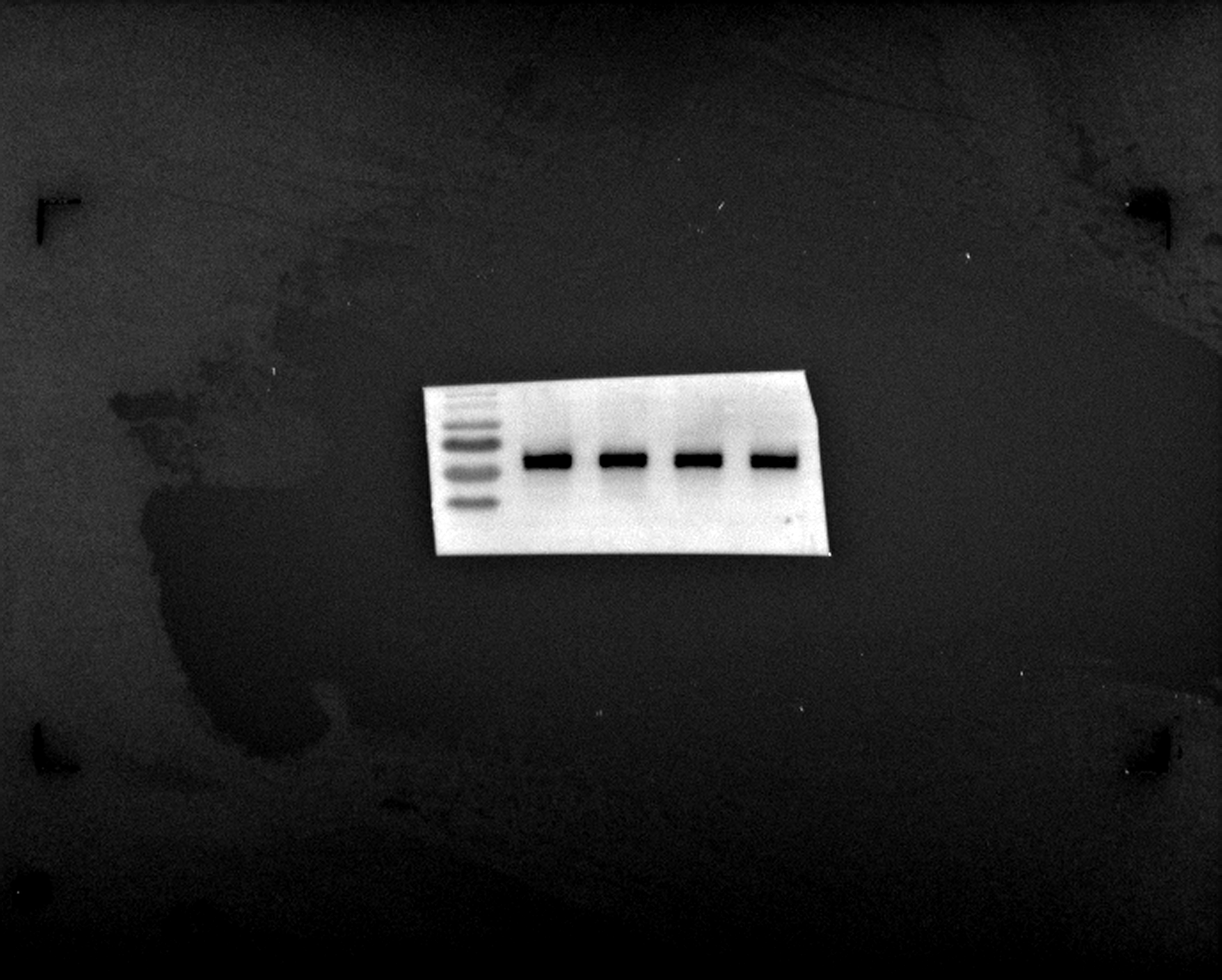

Supplement: Supplemental Material [file KBIE_A_2085390_SM9860.zip › Original Image/Blots Fig5D/p65.Tif]

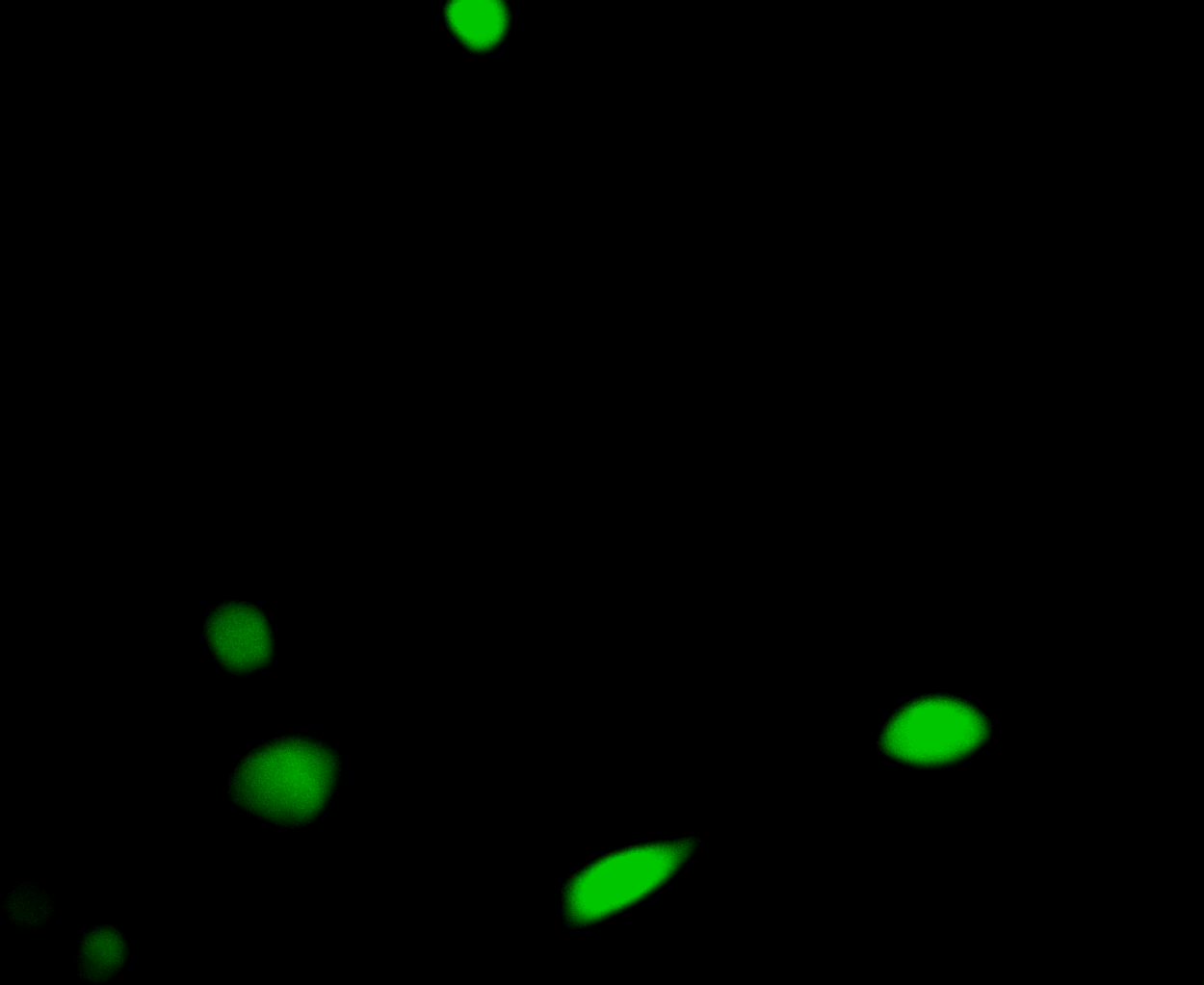

Supplement: Supplemental Material [file KBIE_A_2085390_SM9860.zip › Original Image/Microscopy Fig2A/Apremilast 0μM.png]

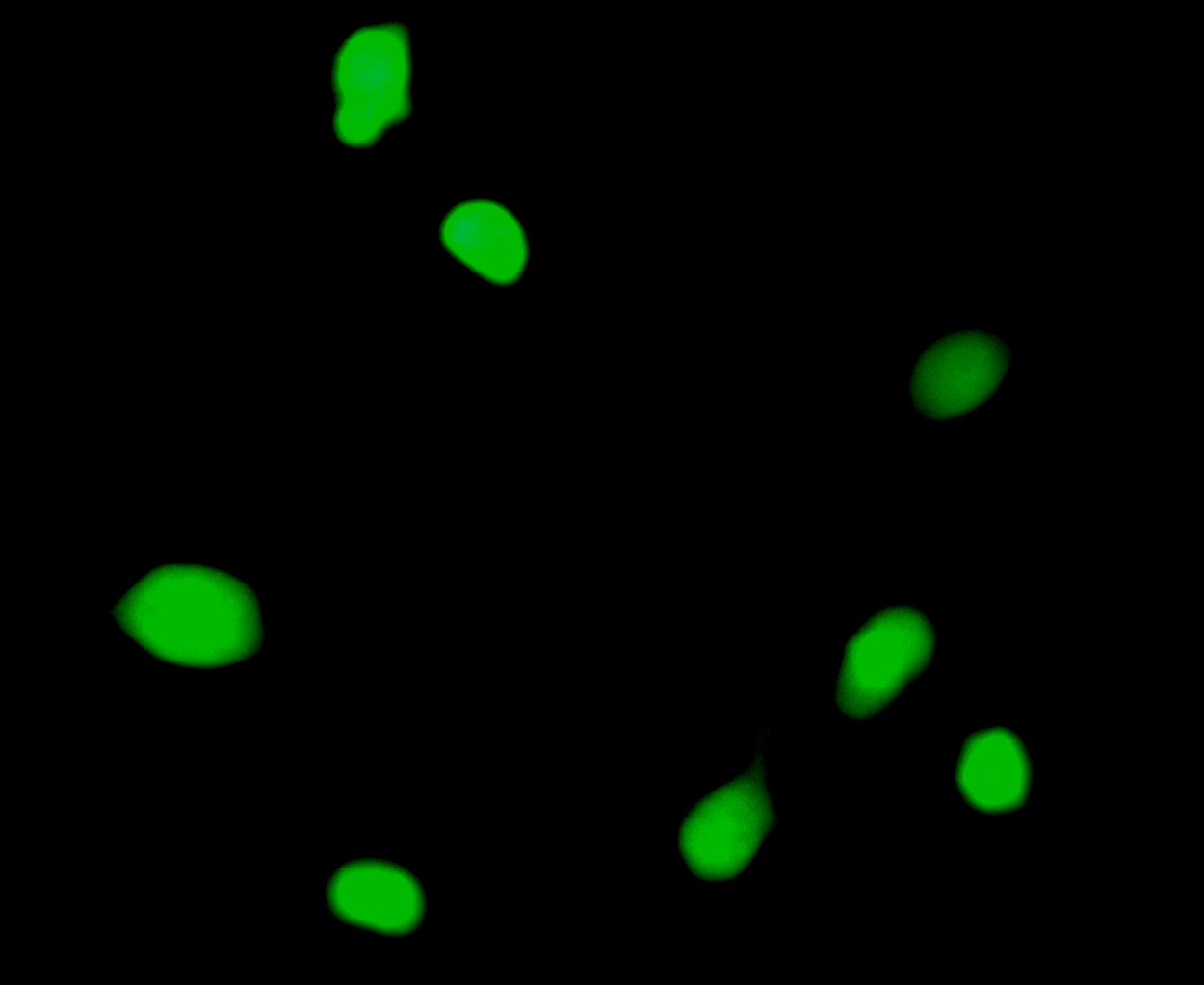

Supplement: Supplemental Material [file KBIE_A_2085390_SM9860.zip › Original Image/Microscopy Fig2A/Ox-LDL+Apremilast 10μM.png]

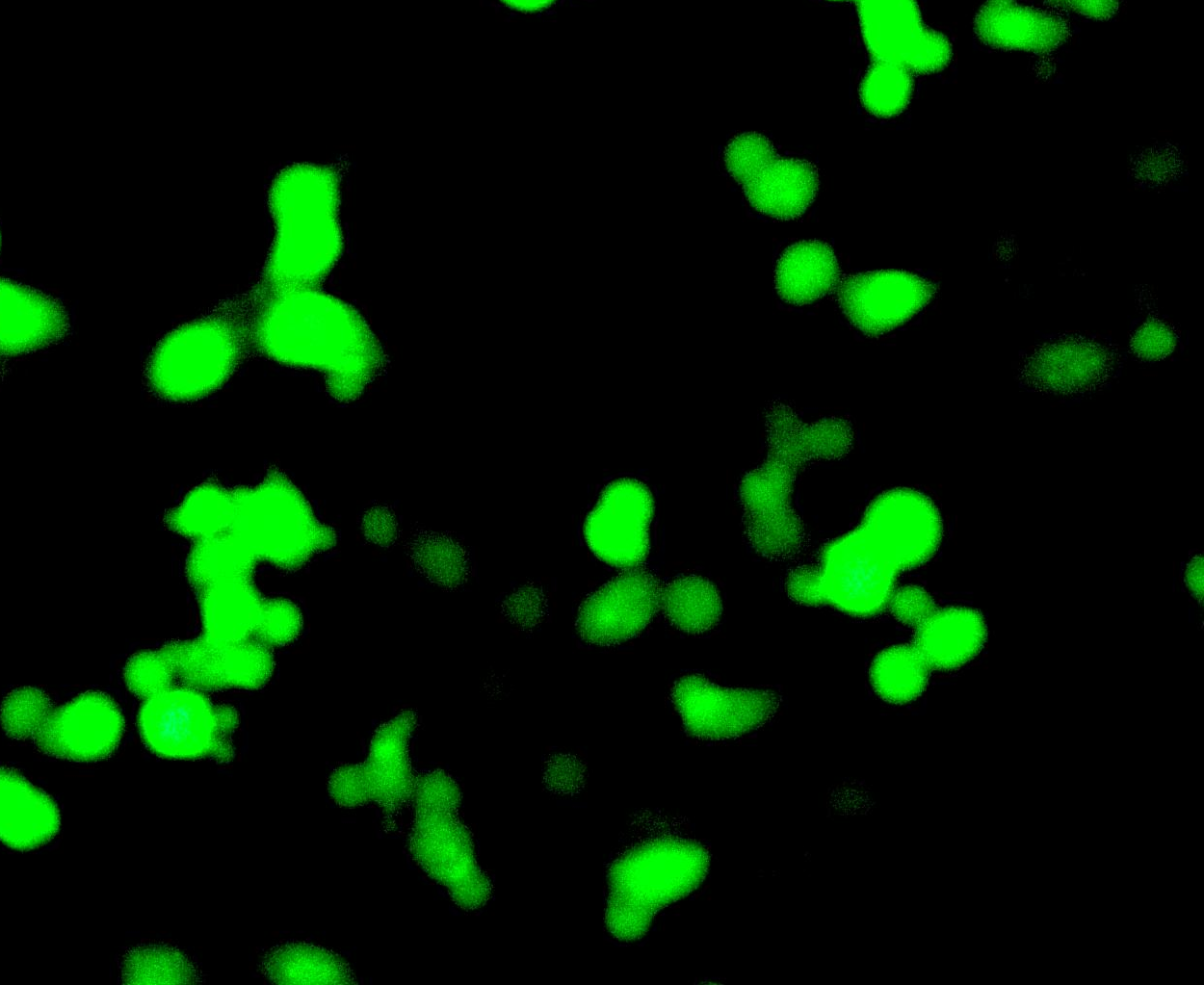

Supplement: Supplemental Material [file KBIE_A_2085390_SM9860.zip › Original Image/Microscopy Fig2A/Ox-LDL+Apremilast 2.5μM.png]

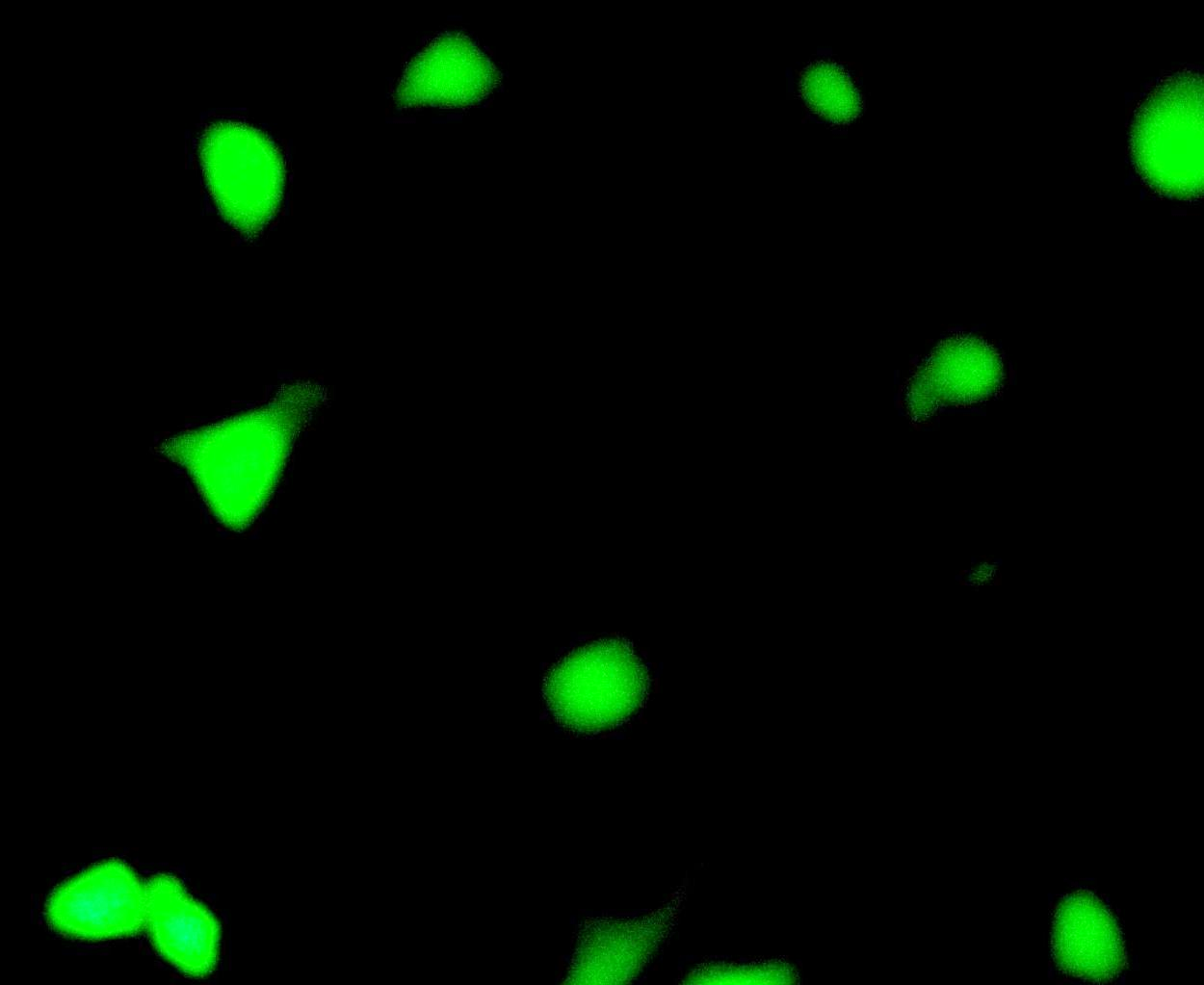

Supplement: Supplemental Material [file KBIE_A_2085390_SM9860.zip › Original Image/Microscopy Fig2A/Ox-LDL+Apremilast 5μM.png]

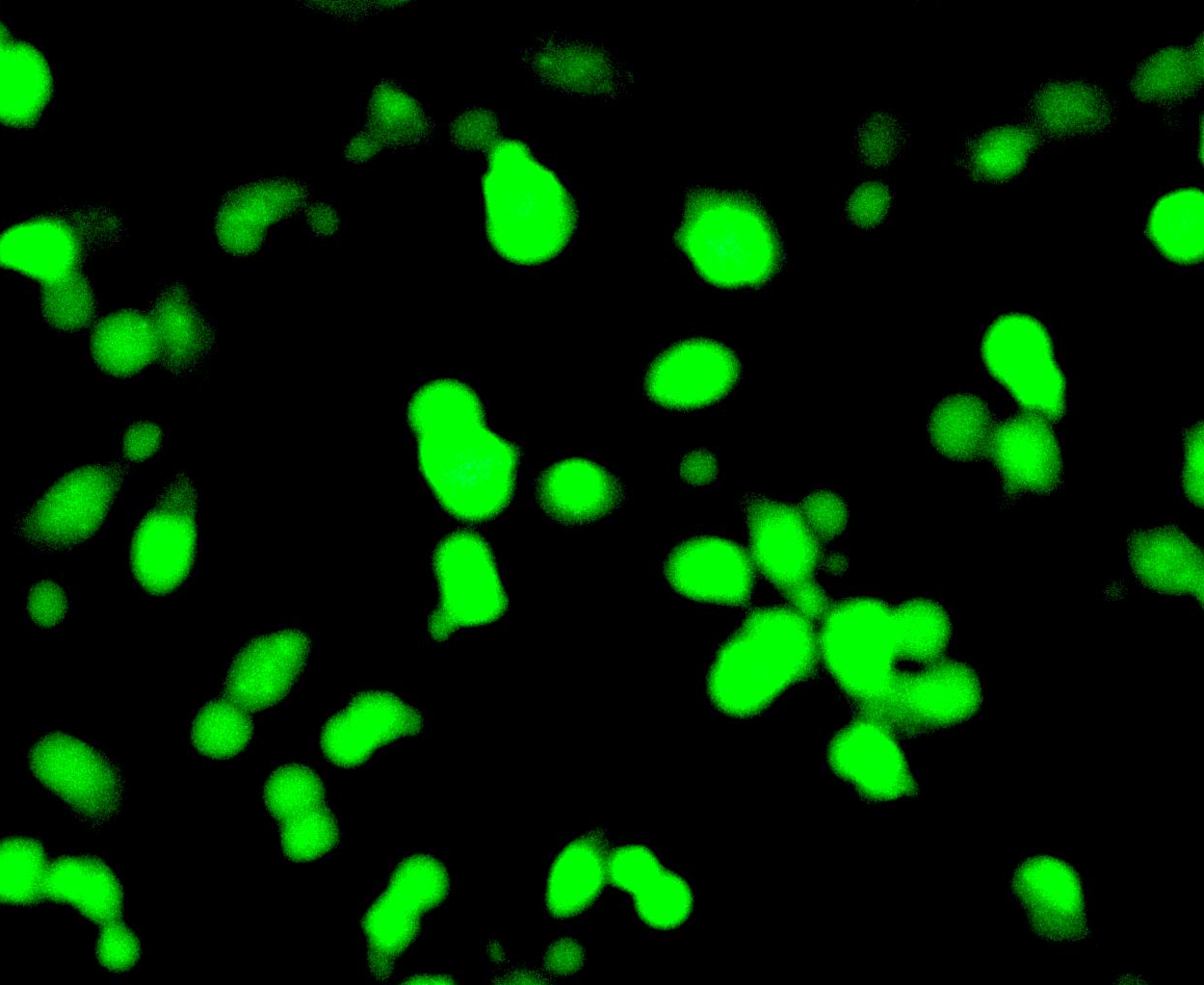

Supplement: Supplemental Material [file KBIE_A_2085390_SM9860.zip › Original Image/Microscopy Fig2A/Ox-LDL.png]

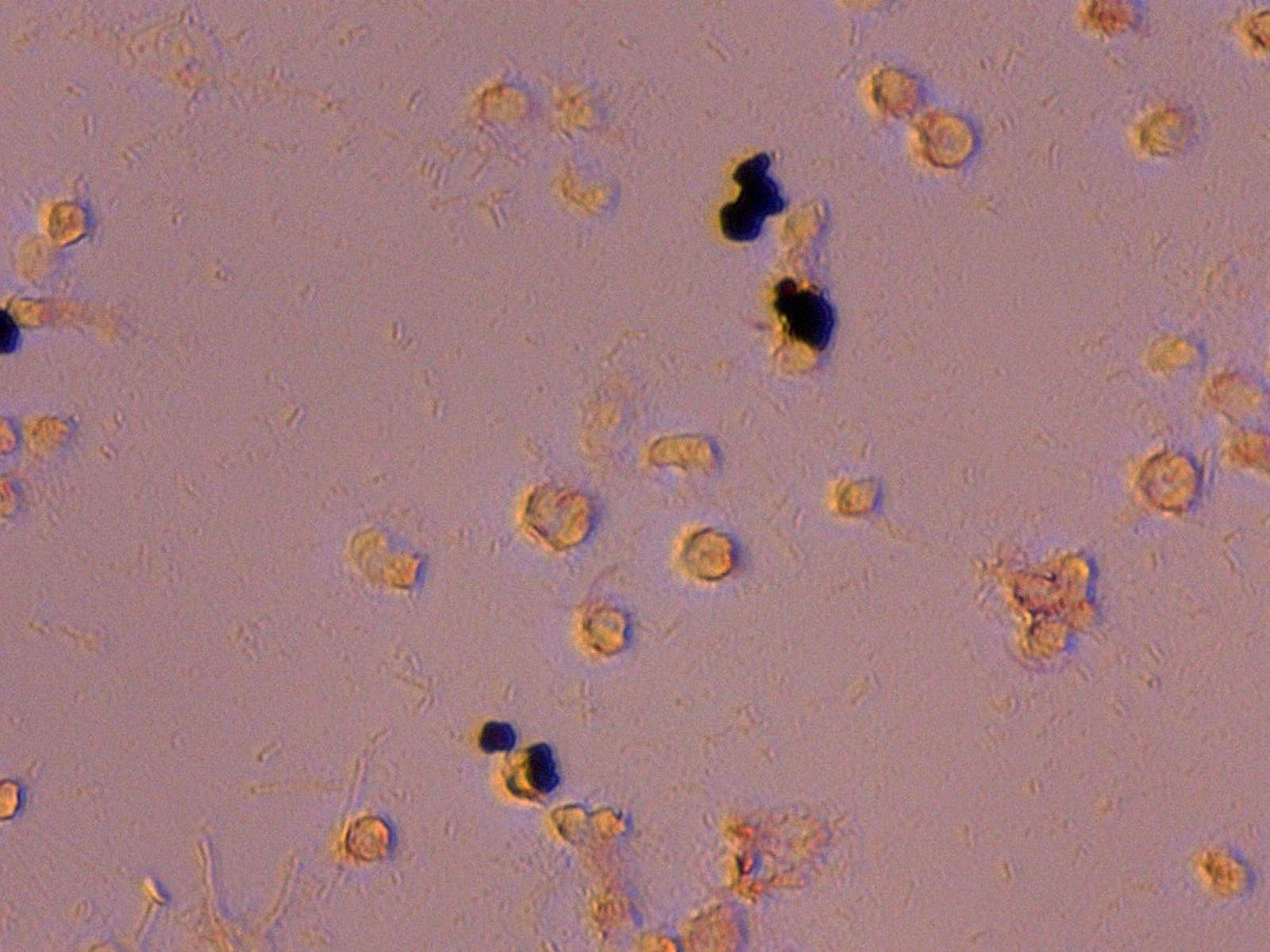

Supplement: Supplemental Material [file KBIE_A_2085390_SM9860.zip › Original Image/Microscopy Fig3A/Control.jpg]

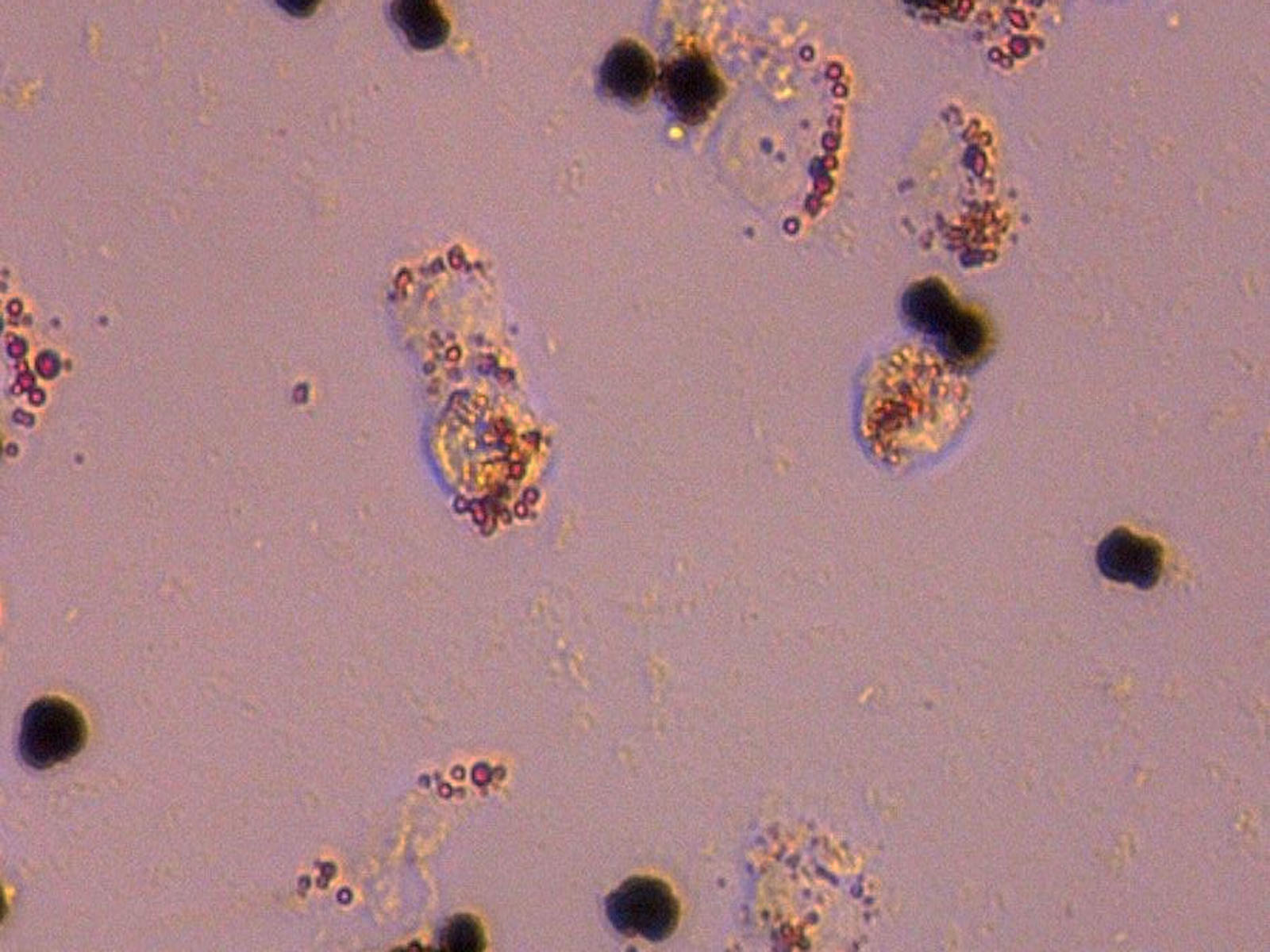

Supplement: Supplemental Material [file KBIE_A_2085390_SM9860.zip › Original Image/Microscopy Fig3A/Ox-LDL+Apremilast 10μM.jpg]

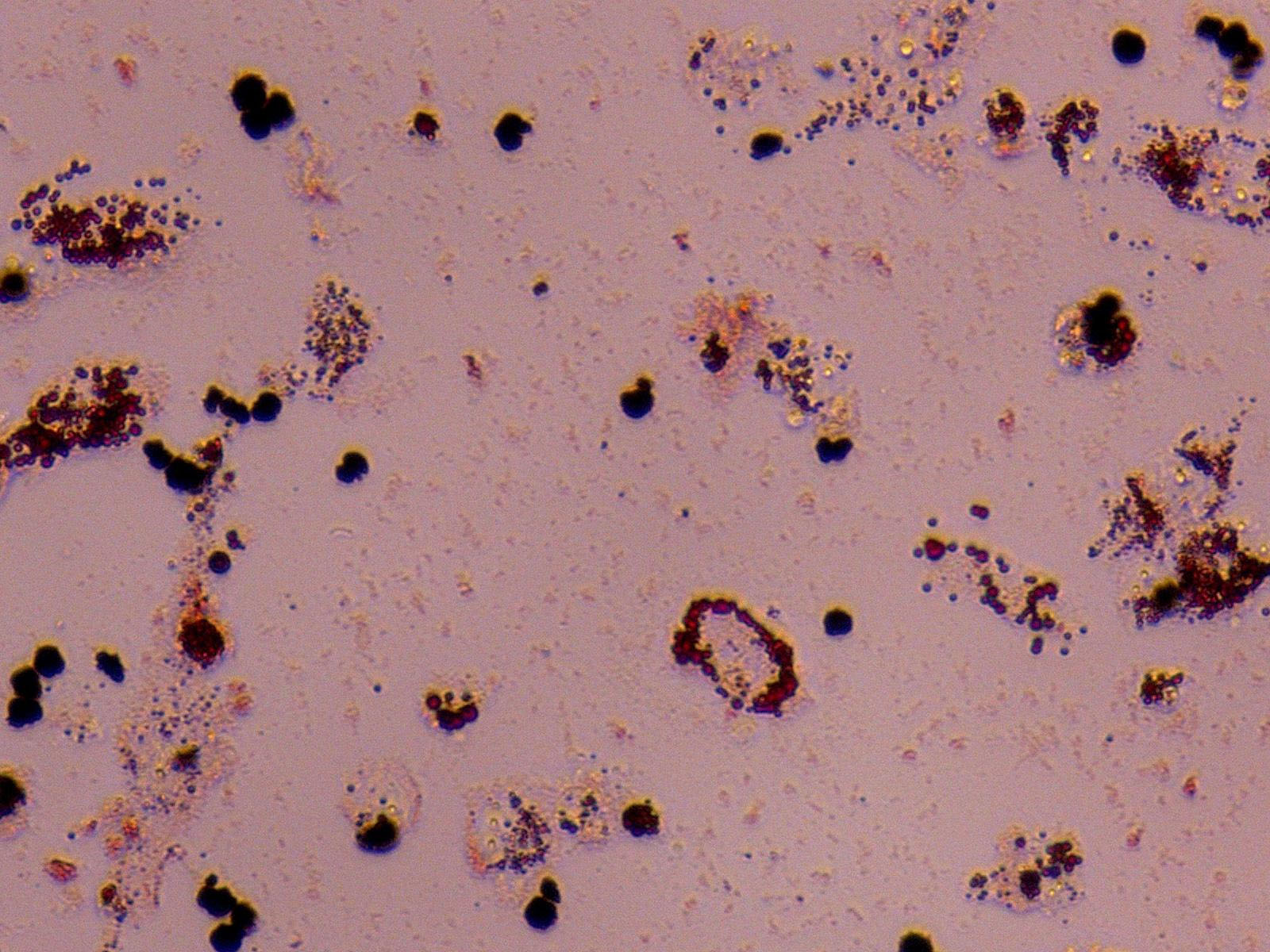

Supplement: Supplemental Material [file KBIE_A_2085390_SM9860.zip › Original Image/Microscopy Fig3A/Ox-LDL+Apremilast 2.5μM.jpg]

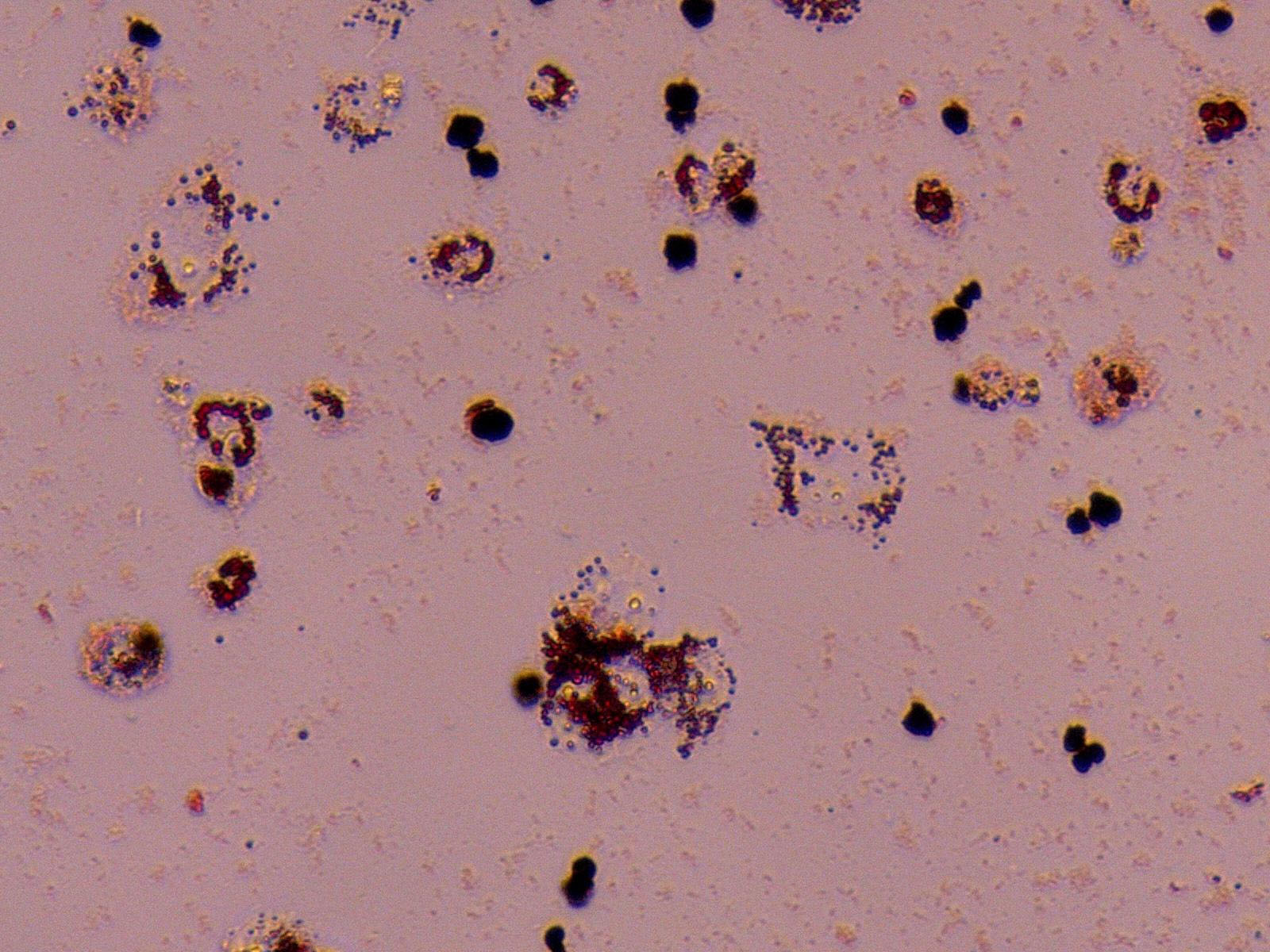

Supplement: Supplemental Material [file KBIE_A_2085390_SM9860.zip › Original Image/Microscopy Fig3A/Ox-LDL+Apremilast 5μM.jpg]

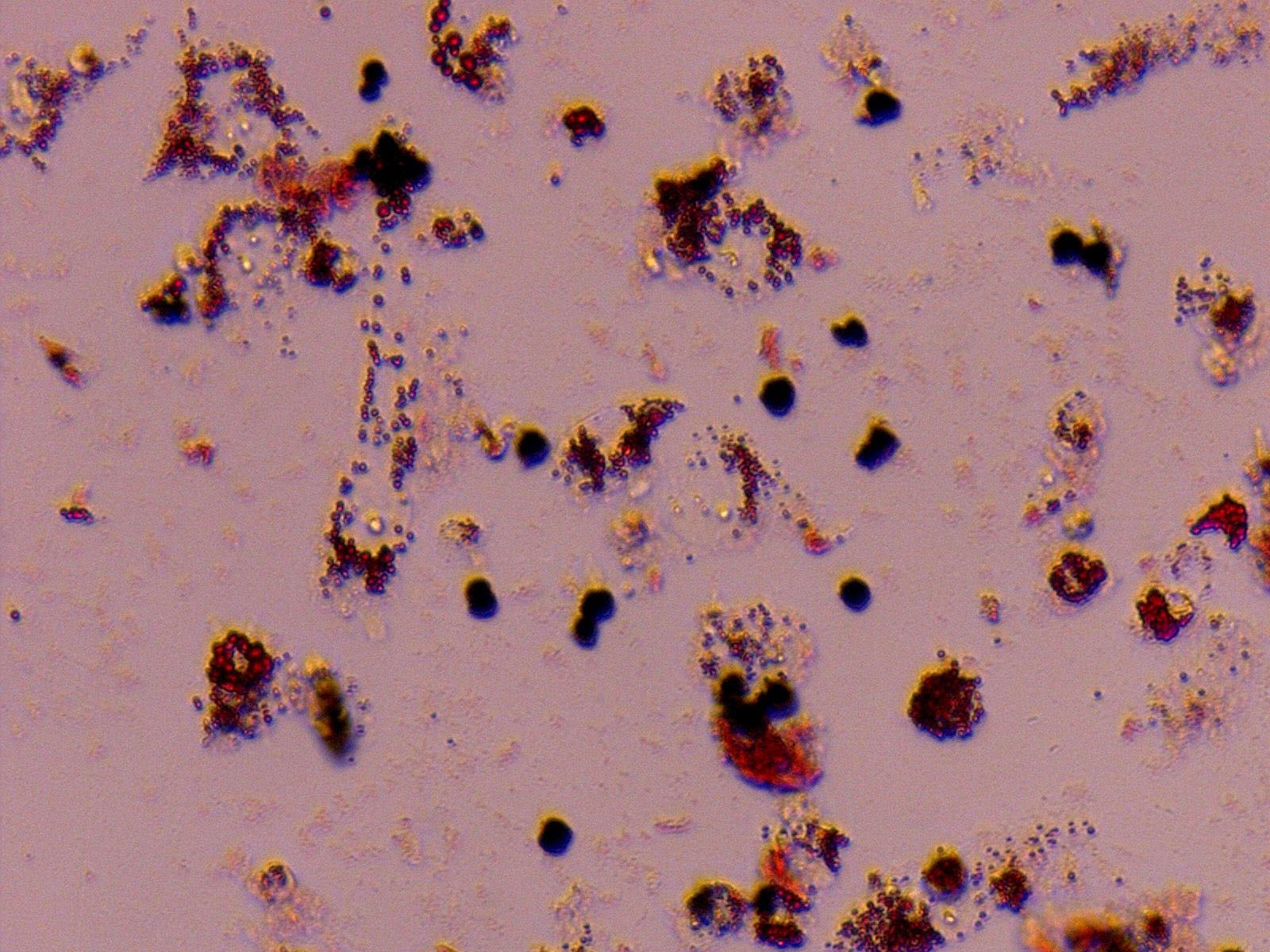

Supplement: Supplemental Material [file KBIE_A_2085390_SM9860.zip › Original Image/Microscopy Fig3A/Ox-LDL.jpg]

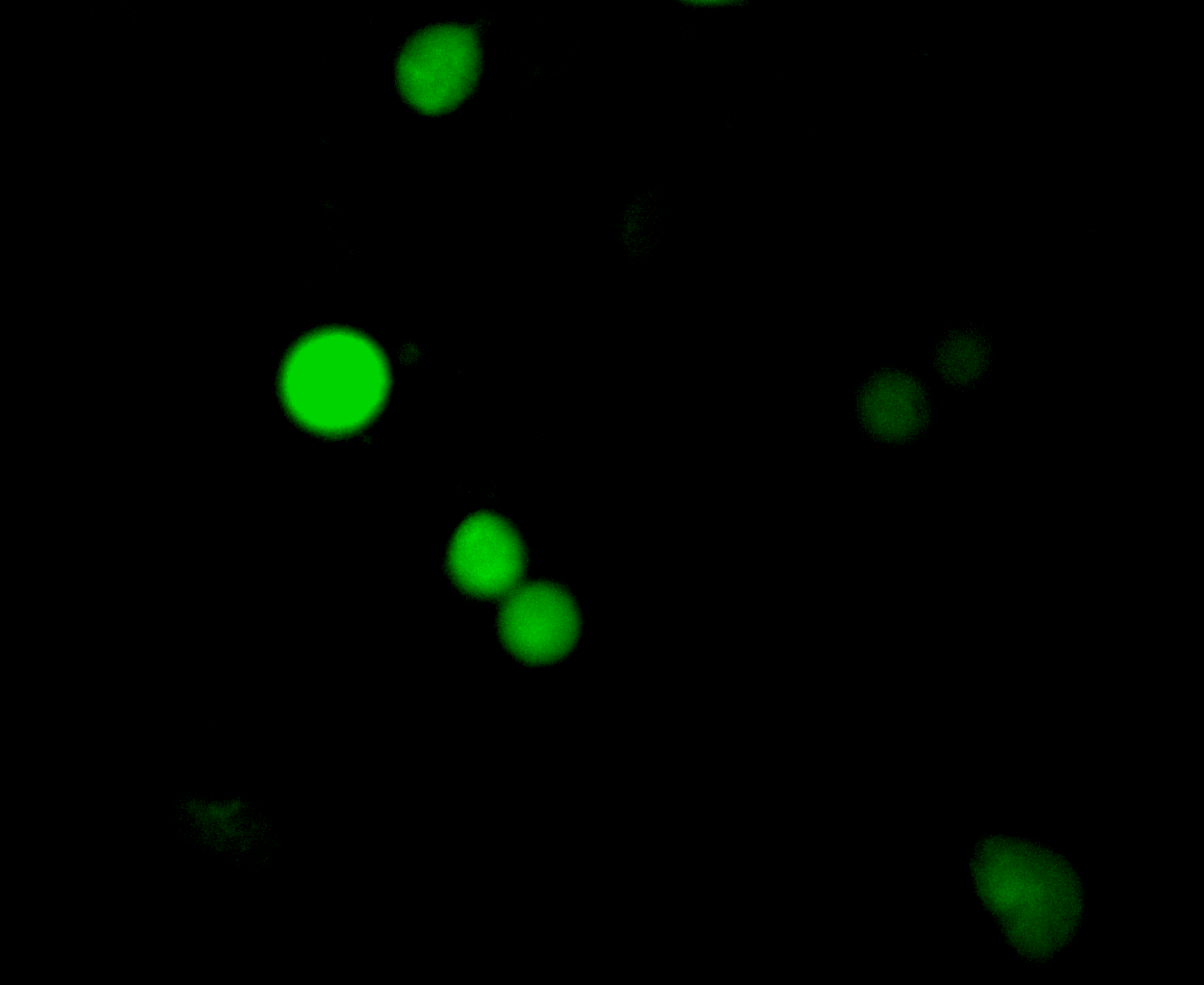

Supplement: Supplemental Material [file KBIE_A_2085390_SM9860.zip › Original Image/Microscopy Fig5A/Control .png]

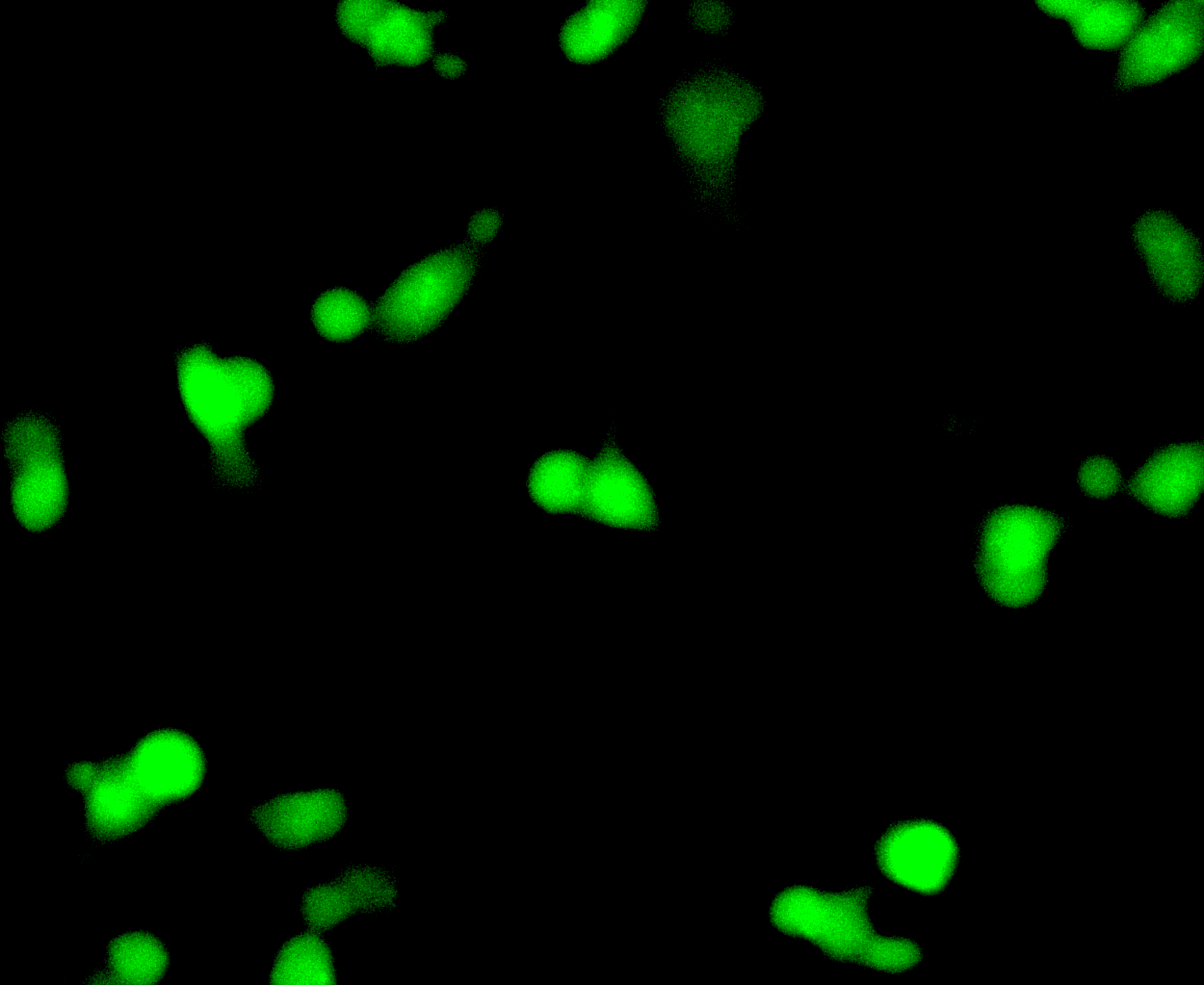

Supplement: Supplemental Material [file KBIE_A_2085390_SM9860.zip › Original Image/Microscopy Fig5A/Ox-LDL+Apremilast 10μM+EX527.png]

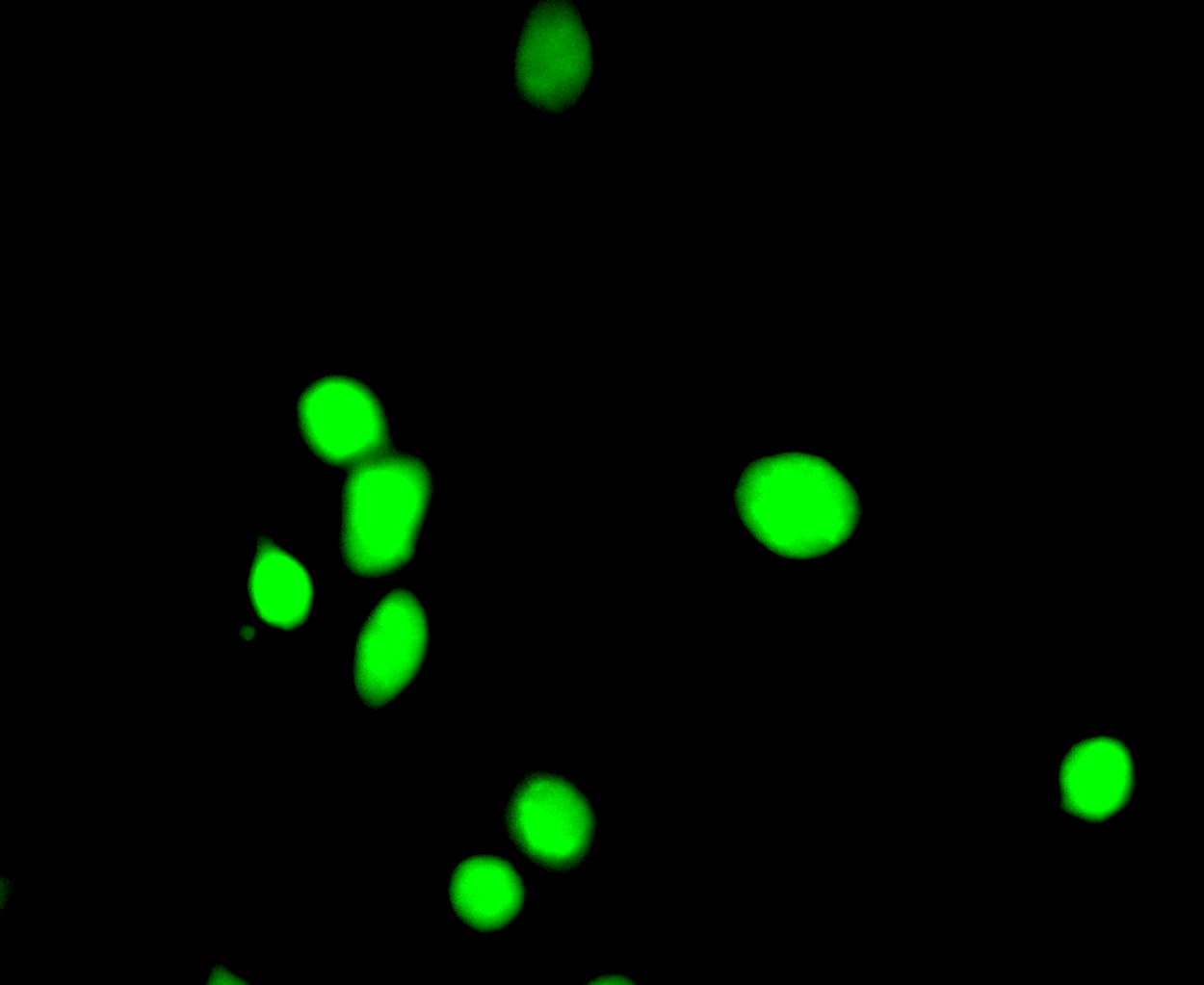

Supplement: Supplemental Material [file KBIE_A_2085390_SM9860.zip › Original Image/Microscopy Fig5A/Ox-LDL+Apremilast 10μM.png]

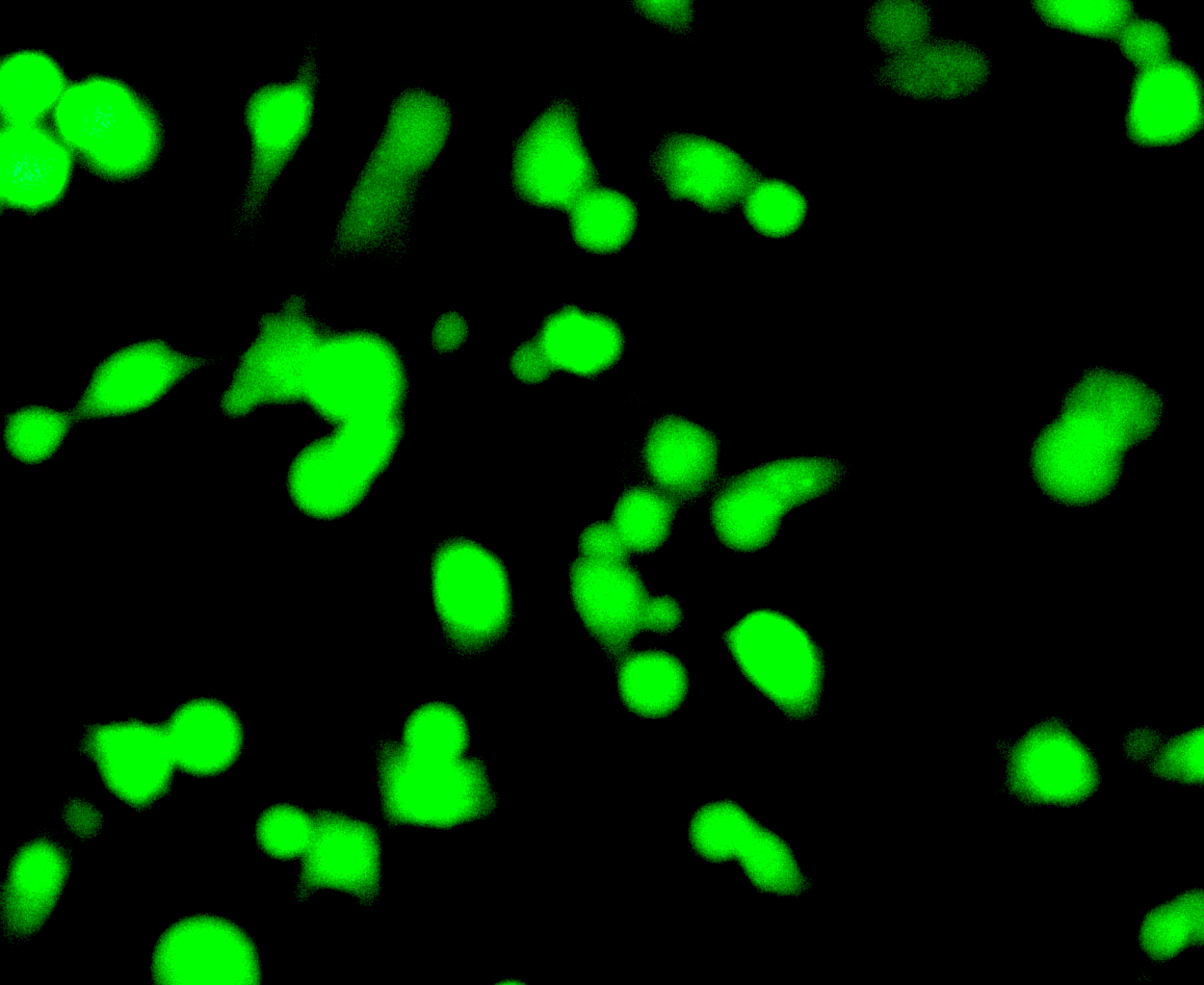

Supplement: Supplemental Material [file KBIE_A_2085390_SM9860.zip › Original Image/Microscopy Fig5A/Ox-LDL.png]

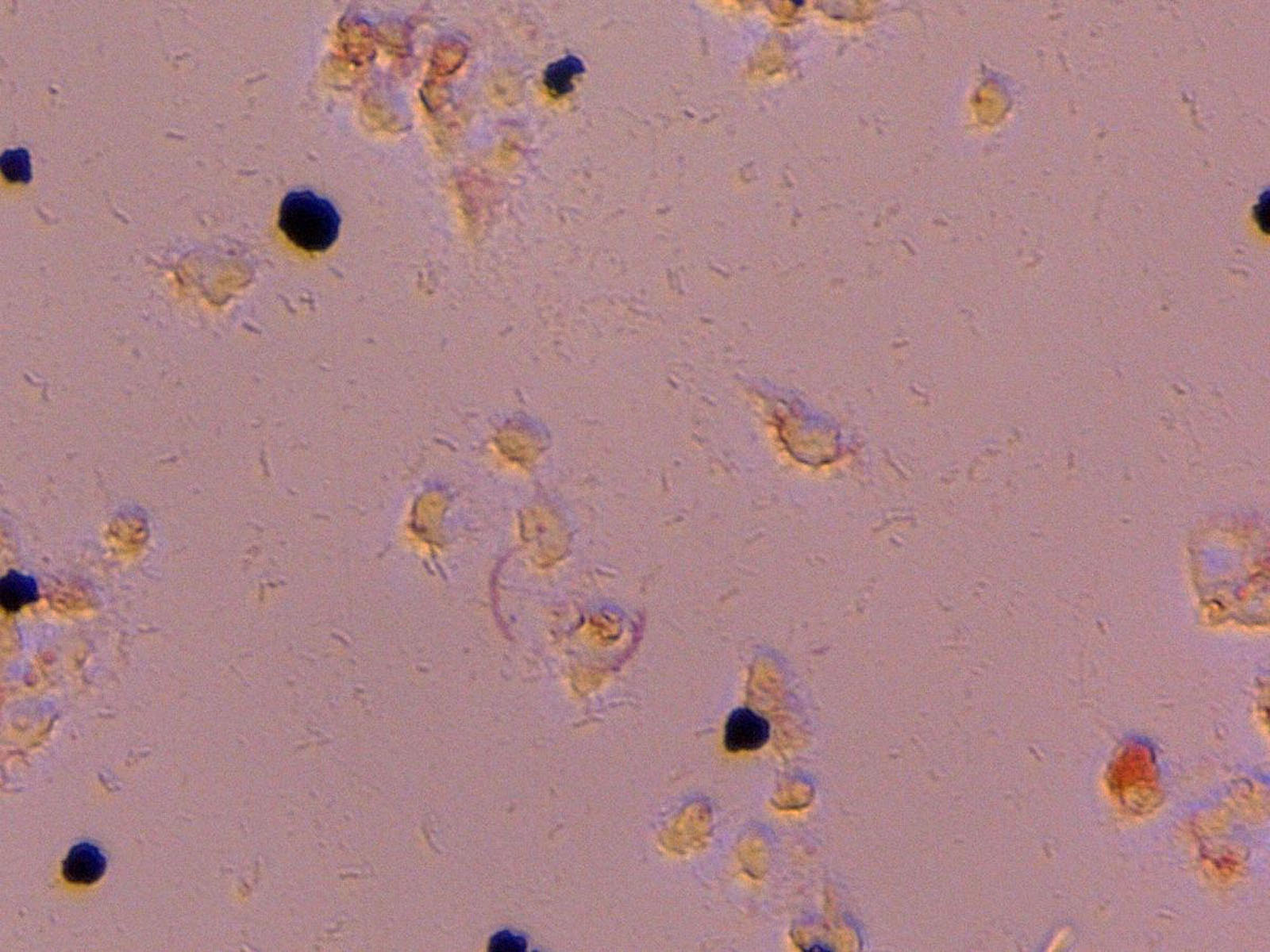

Supplement: Supplemental Material [file KBIE_A_2085390_SM9860.zip › Original Image/Microscopy Fig6A/Control .jpg]

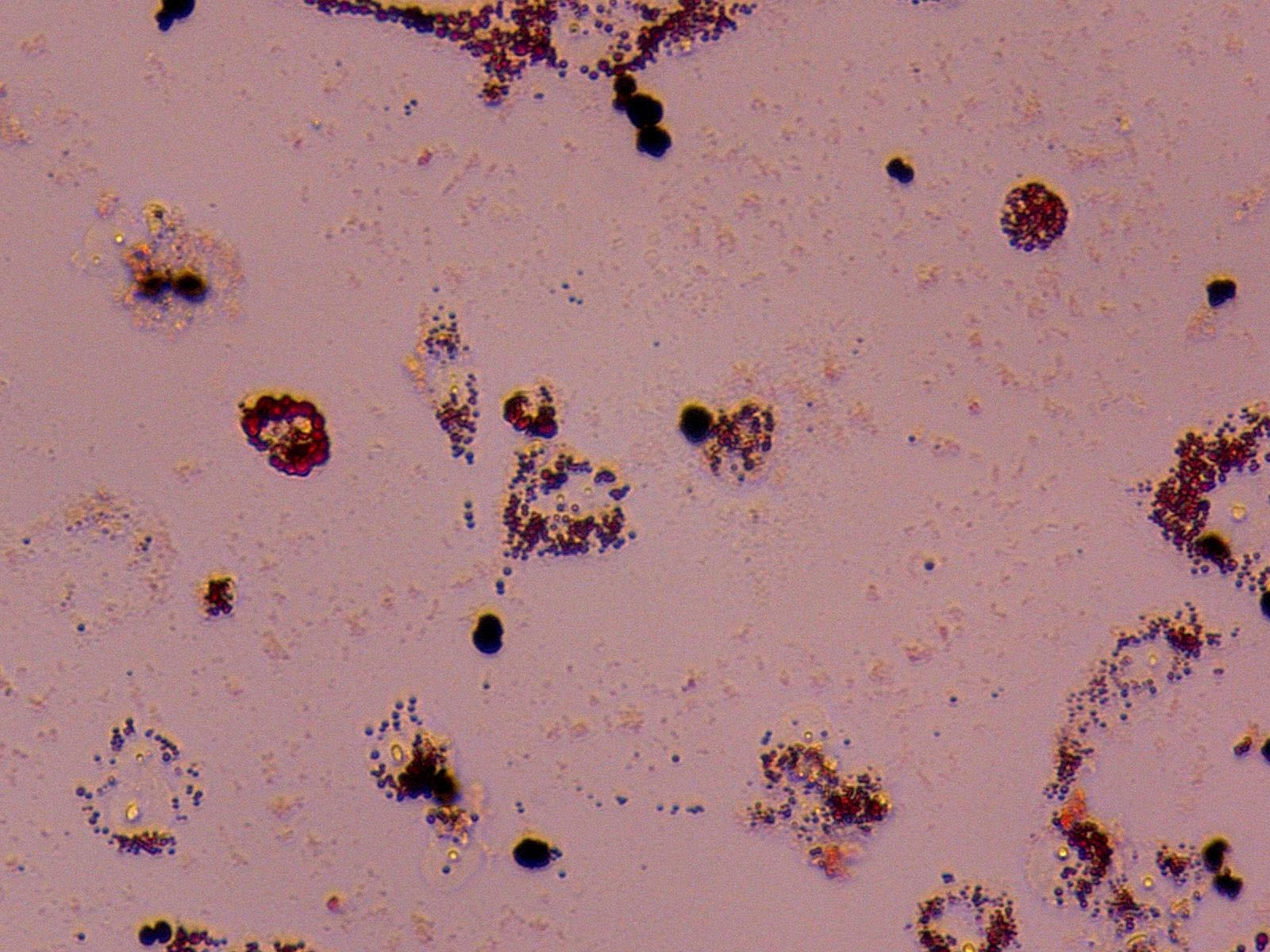

Supplement: Supplemental Material [file KBIE_A_2085390_SM9860.zip › Original Image/Microscopy Fig6A/Ox-LDL+Apremilast 10μM+EX527.jpg]

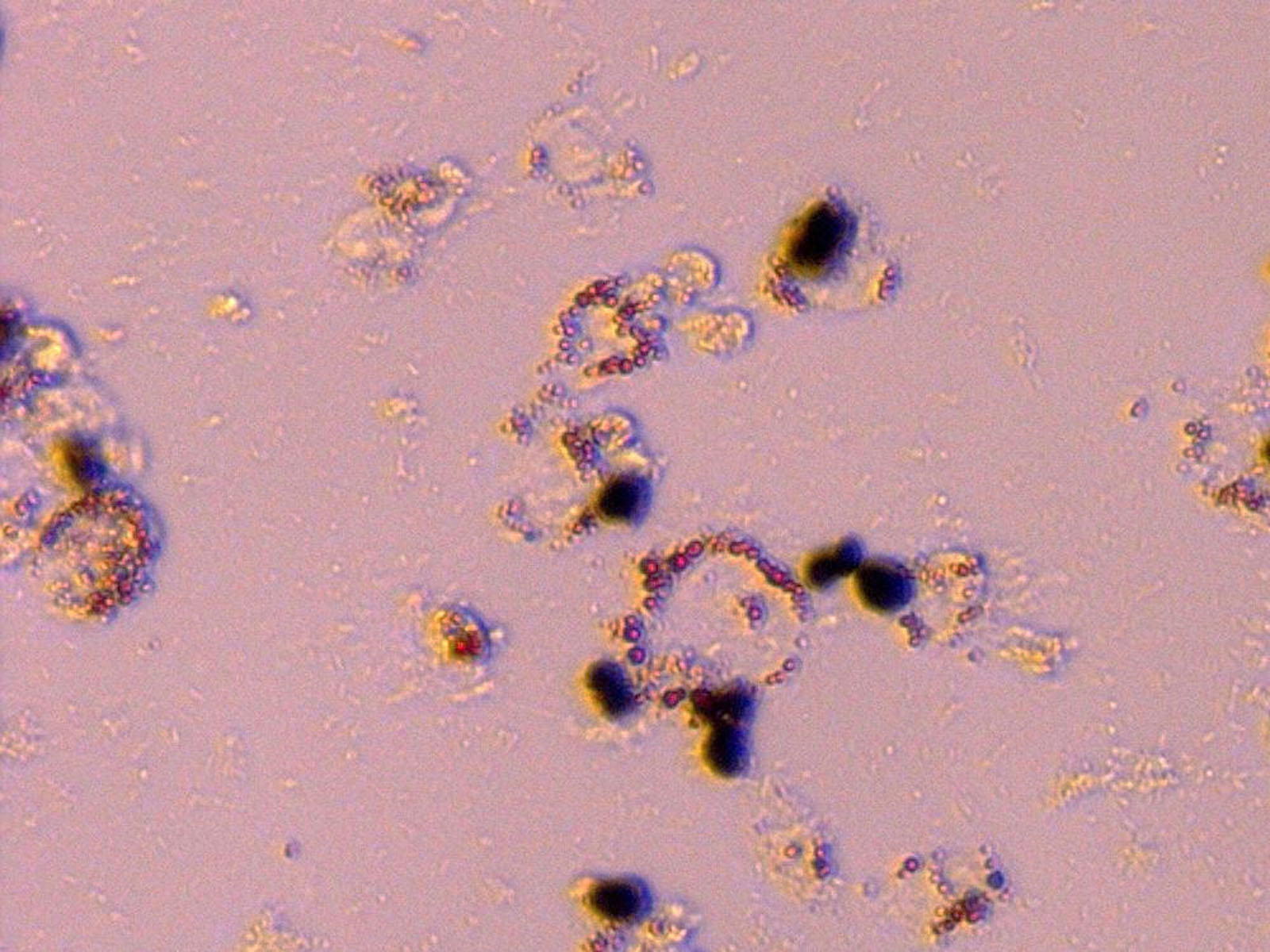

Supplement: Supplemental Material [file KBIE_A_2085390_SM9860.zip › Original Image/Microscopy Fig6A/Ox-LDL+Apremilast 10μM.jpg]

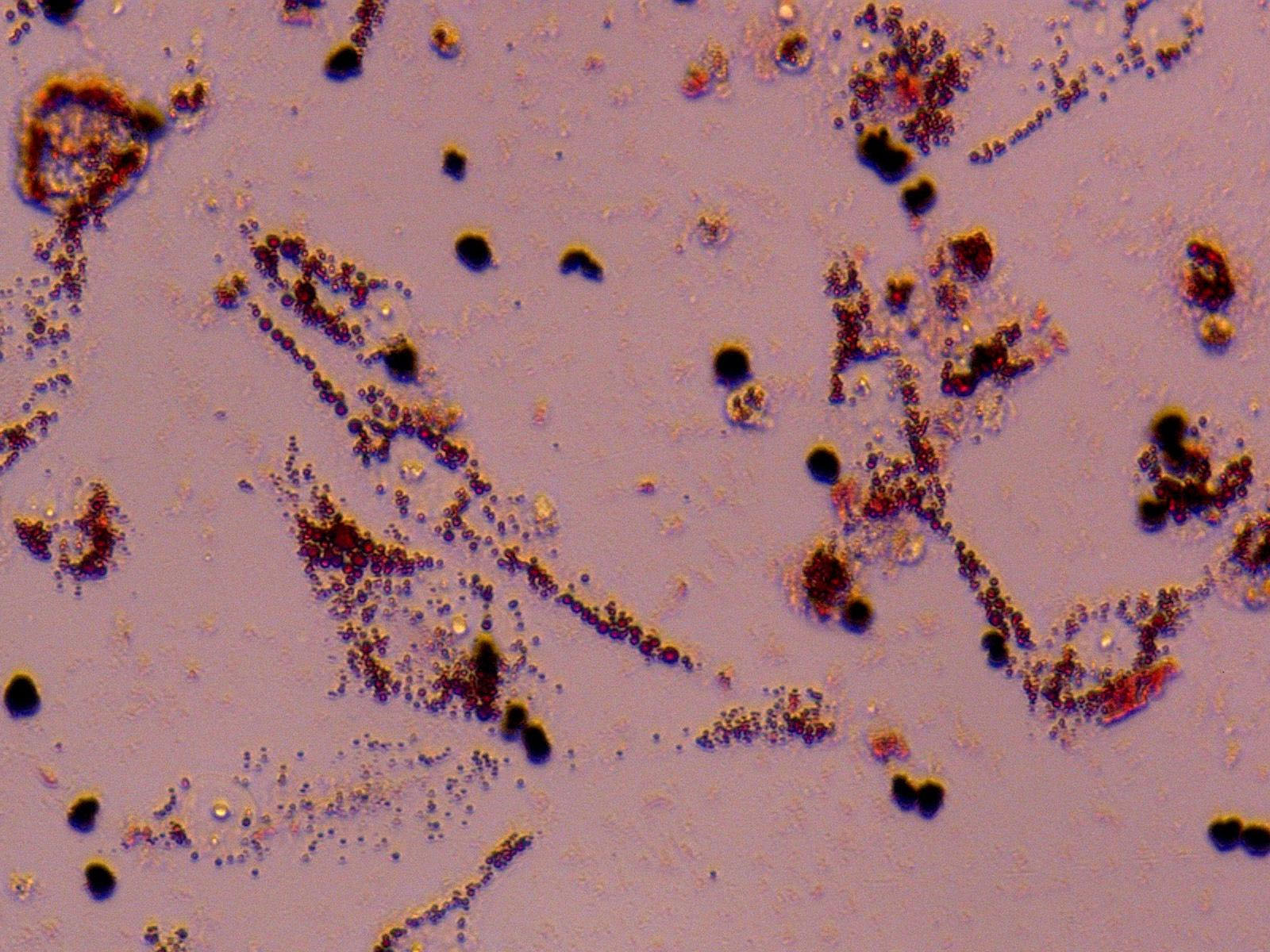

Supplement: Supplemental Material [file KBIE_A_2085390_SM9860.zip › Original Image/Microscopy Fig6A/Ox-LDL.jpg]
